# Discovery of Isoquinolinoquinazolinones as a Novel Class of Potent PPARγ Antagonists with Anti-adipogenic Effects

**DOI:** 10.1038/srep34661

**Published:** 2016-10-03

**Authors:** Yifeng Jin, Younho Han, Daulat Bikram Khadka, Chao Zhao, Kwang Youl Lee, Won-Jea Cho

**Affiliations:** 1 College of Pharmacy and Research Institute of Drug Development, Chonnam National University, Gwangju 61186, Republic of Korea

## Abstract

Conformational change in helix 12 can alter ligand-induced PPARγ activity; based on this reason, isoquinolinoquinazolinones, structural homologs of berberine, were designed and synthesized as PPARγ antagonists. Computational docking and mutational study indicated that isoquinolinoquinazolinones form hydrogen bonds with the Cys285 and Arg288 residues of PPARγ. Furthermore, SPR results demonstrated strong binding affinity of isoquinolinoquinazolinones towards PPARγ. Additionally, biological assays showed that this new series of PPARγ antagonists more strongly inhibit adipocyte differentiation and PPARγ2-induced transcriptional activity than GW9662.

The incidence of metabolic syndromes, including diabetes and heart disease, is increasing worldwide, and this has led to extensive research into adipogenesis. A ligand-dependent nuclear receptor, peroxisome proliferator-activated receptor γ (PPARγ), is the key regulator of adipogenesis^1^. PPARγ is highly conserved across all species and expressed predominantly in adipose tissue, macrophages, colon epithelium, and in skeletal muscle. PPARγ regulates gene expression related to adipogenesis and glucose metabolism. The PPARγ isoforms (PPARγ1, PPARγ2, and PPARγ3) are functionally identical; however, a recent report indicates that PPARγ2 is the principal regulator of adipogenesis^2^. As a result, PPARγ2 is a potential therapeutic target for type 2 diabetes mellitus, dyslipidemia, atherosclerosis, obesity, and other metabolic diseases^3^,^4^.

PPARγ agonists have been used to treat metabolic diseases for decades. Rosiglitazone **1**, an example of a thiazolidinedione (TZD) PPARγ agonist, is an insulin-sensitizing agent (Fig. 1A). However, the limitations and side-effects of TZDs, such as edema, weight gain, and increased incidence of heart attack, discouraged further development and prevented clinical application of TZD-based PPARγ agonists^5^. Thus, the development of novel agents that modulate PPARγ is required.

It has been reported that inhibition of PPARγ activity can also improve insulin sensitivity^6^. Interestingly, the PPARγ antagonist, SR-202 **2**, shows antiobesity and antidiabetic effects, and lacks the adverse effects caused by PPARγ agonists (Fig. 1A)^7^. A well-known PPARγ antagonist, GW9662 **3**, was identified in a competition-binding assay against the human ligand-binding domain (region E/F) of PPARγ. GW9662 has high binding affinity, and shows potential inhibitory activity towards PPARγ^8^. Berberine **4**, a tetracyclic isoquinoline alkaloid, has been reported to suppress adipocyte differentiation in 3T3-L1 cells by inhibiting PPARγ and increasing insulin sensitivity^9^. Thus, the promising results of PPARγ antagonists led us to discover a novel class of agents that could be used to treat PPARγ-related diseases.

Usually, nuclear receptors regulate gene transcription by binding to DNA in conjunction with a variety of cofactors^10^. The binding site of cofactors, the activation function-2 (AF-2) region, is altered by a conformational change in helix 12 (H12). H12 structure-function models of nuclear receptor ligand binding domains (LBDs) have shown that, at the molecular level, ligand-modulated agonism and antagonism depends on the conformation of H12. In the case of PPARγ, it has been shown that agonists can stabilize the ligand-binding pocket through interaction with H12^11^,^12^. The 3D structure of the complex that is formed between PPARγ and the agonist rosiglitazone **1** contains a hydrogen bond between a nitrogen atom in rosiglitazone and the hydroxyl group of Tyr473, which lies in H12 (PDB: 2PRG)^13^. This interaction helps rosiglitazone stabilize conformational changes in PPARγ, particularly in the transcription cofactor-binding AF-2 region of H12^14^. In contrast, a PPARγ antagonist, GW9662 (in non-covalent complex with PPARγ, PDB: 3E00) does not have any interaction with H12^15^.

The LBD of nuclear receptors that contains the AF-2 region, is the primary site investigated for drug discovery. Our research group has succeeded in designing androgen receptor antagonists, nicotinamides, and demonstrated that the antagonist effect of these analogues is a result of their effect on the conformation of H12; agonists lock the conformation of H12 giving a closed conformation of ligand binding pocket (LBP), while antagonists give an open conformation of LBP^16^. On the basis of this principle, we investigated and synthesized isoquinolinoquinazolinones as a novel class of PPARγ antagonists. Compared with well-known PPARγ antagonists, such as GW9662, isoquinolinoquinazolinones which resemble berberine can be expected to possess more drug-like characteristics. Herein, we report a new series of PPARγ antagonists, which is much more potent than GW9662 according to biological evaluations.

## Drug Design

We have previously reported the modification of protoberberines by altering the ring size or introducing a heteroatom into ring B^17^,^18^,^19^,^20^,^21^. In order to investigate a new series of PPARγ antagonists, we initially focused on 5-oxaprotoberberines, a class of berberine bioisosteres. The oxaprotoberberines affected adipogenesis; however, the activity was not better than berberine (Table 1, **10a–h**). For an effective rational design strategy for PPARγ antagonists, molecular modeling was used to study the interaction between oxaprotoberberines and the GW9662 binding pocket of the PPARγ-GW9662-RXRα-retinoic acid-NCoA-2-DNA complex (PDB: 3E00)^15^.

**Figure 1:**
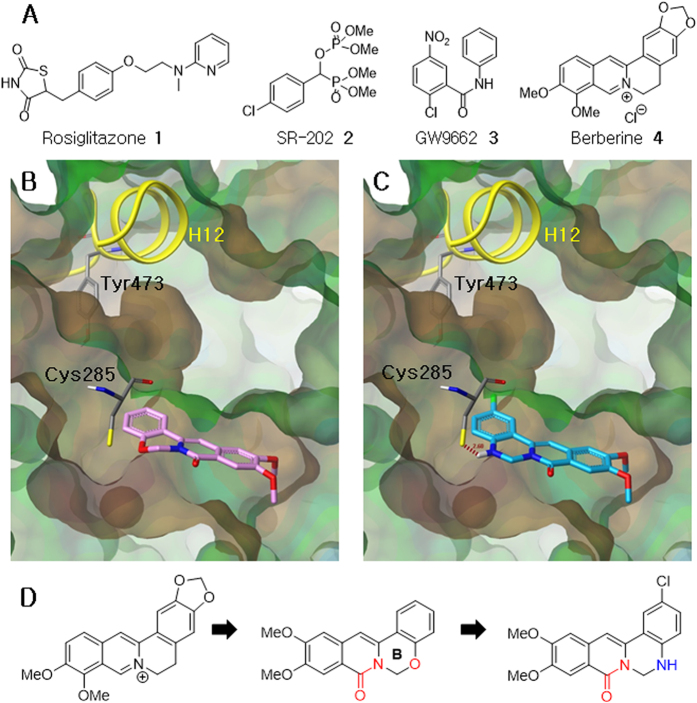
Known PPARγ agonists and antagonists, molecular docking modes and drug design. (**A**) Rosiglitazone **1**, SR-202 **2**, GW9662 **3**, and Berberine **4**. (**B**) Docking mode of 5-oxaprotoberberine (pink) in the LBP of PPARγ. (**C**) Docking mode of isoquinolinoquinazolinone (blue) in the active site of PPARγ. (**D**) Design of isoquinolinoquinazolinones.

Oxaprotoberberines, as shown in Fig. 1B, do not interact with H12, and the tetracyclic core is positioned in a hydrophobic region of the pocket. In addition, the oxygen atom in ring B is close to Cys285; this gave rise to an idea of exchanging the O atom with -NH, to generate isoquinolinoquinazolinones (Fig. 1C,D).

The binding mode between isoquinolinoquinazolinones and PPARγ indicates that the new amino group is located exactly where it was predicted, interacts through hydrogen-bonding to the Cys285 amino acid residue on helix 3 of PPARγ, and does not affect the conformation of H12 (Fig. 1C). These results provided an incentive to investigate the effects of isoquinolinoquinazolinones on PPARγ inhibition and adipocyte differentiation.

## Results and Discussion

The isoquinolinoquinazolinones **8a-o** were synthesized using a strategy that was similar to our previously reported synthesis method of oxaprotoberberine^20^. The synthesis of isoquinolinoquinazolinones **8** involved a cycloaddition reaction between lithiated toluamides and 2-aminobenzonitriles (Scheme S1). The *o*-toluamides **5** were deprotonated using *n*-BuLi and reacted with the 2-aminobenzonitriles **6** to give the 3-arylisoquinolones **7**. Finally, intramolecular cyclization was performed using diiodomethane and K_2_CO_3_ as base. Furthermore, **8o** was reduced with lithium aluminium hydride to give the dihydro derivative **9** (Scheme S2).

**Figure 2:**
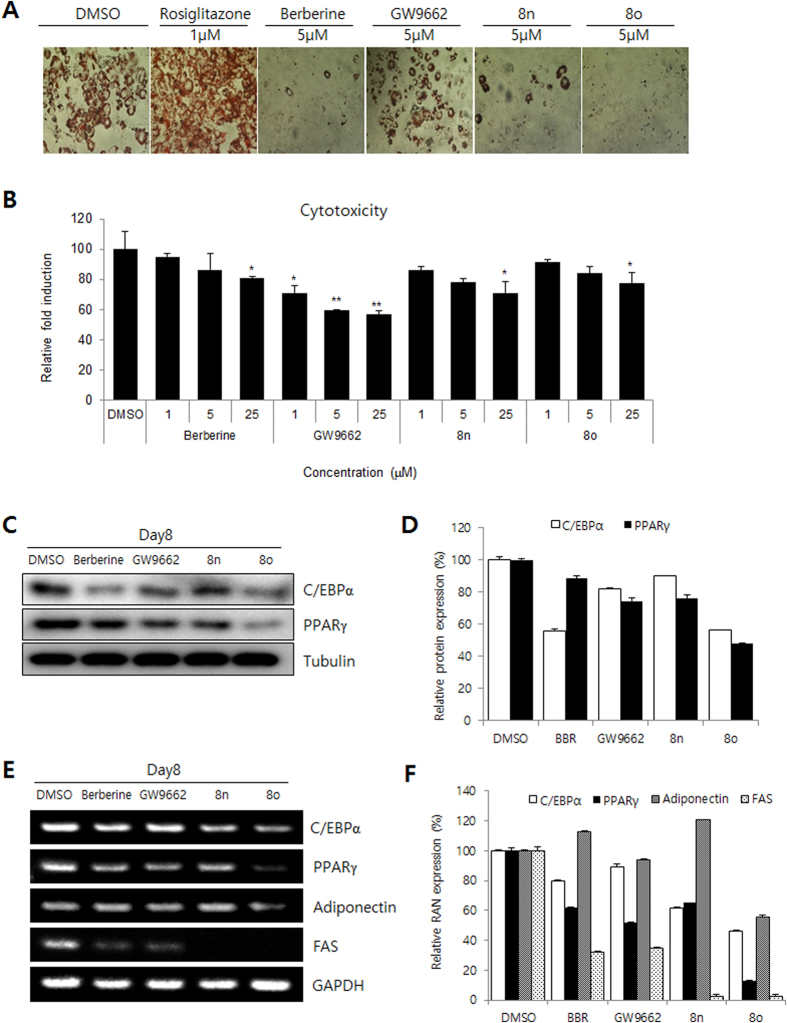
Effect of isoquinolinoquinazolinones treatment on 3T3-L1 cells *in vitro*. (**A**) Oil Red O staining assay. (**B**) Cytotoxicity of berberine, GW9662, **8n**, and **8o** on 3T3-L1 cells. Data show mean ± SD of at least three experiments. ^*^P < 0.05 and ^**^P < 0.01 as compared to DMSO control. (**C**,**D**) Inhibitory activity of berberine, GW9662, **8n**, and **8o** as evidenced by protein level of adipogenesis markers. (**E**,**F**) Inhibitory activity of berberine, GW9662, **8n**, and **8o** as evidenced by RNA level of adipogenesis markers.

The GW9662-mediated inhibition of PPARγ was confirmed using an Oil Red O staining assay to measure adipocyte differentiation^8^. To investigate whether the novel compounds could inhibit adipocyte differentiation, 3T3-L1 preadipocytes were incubated with differentiation medium (MDI; insulin, dexamethasone, and isobutyl methyl xanthine) in the presence of increasing concentrations of isoquinolinoquinazolinones. Adipogenesis was analyzed using Oil Red O staining after treatment with the compounds. Most of the compounds showed potential inhibitory activity towards adipocyte differentiation (Fig. 2A and Table 1).

A brief structure and activity relationship (SAR) study was performed in the context of adipocyte differentiation inhibition. The different substituents in ring D affect the inhibitory activity of 5-oxaprotoberberines **10**. Compounds that contained a methyl group at both the C10 and C11 positions or a methoxy group at both the C11 and C12 positions (**10c** and **10d**) were more active than those with a single methyl group at C11 or C12 (**10a** and **10b**). Installation of a methylene dioxyl group across the C2 and C3 positions in ring A also increased the inhibitory activities (**10e**, 44.8% and **10f**, 30.5%). In the case of isoquinolinoquinazolinones, introducing a single methyl group at C10 (**8f** and **8g**) or a methyl group at both C10 and C11 (**8h-j**) decreased the activity. Introducing a chlorine substituent at C2 or C3 (**8e, 8g, 8i, 8j**) can increase the inhibitory activity. Among all the compounds tested, the isoquinolinoquinazoline compounds that had methoxy groups at both C10 and C11 and a chlorine substituent on ring A (**8n** and **8o)** inhibited adipocyte differentiation with the greatest potency (71.5% and 82.4%, respectively). The mechanisms of action of these two compounds were further investigated.

Prior to testing the PPARγ antagonist potential of isoquinolinoquinazolinones, their cytotoxicity in 3T3-L1 cells (normal cells) was examined. At a concentration of 25 μM, compounds **8n** and **8o** exhibited little or no cytotoxic effect (>70% cell viability; Fig. 2B). These two compounds were then tested for PPARγ inhibitory activity.

PPARγ alone or together with CCAAT-enhancer-binding protein α (C/EBPα) regulates many adipocyte genes that are involved in creating and maintaining the adipocyte phenotype^22^. To further analyze the effect of isoquinolinoquinazolinones on adipogenesis, the expression of the adipocyte marker genes (*C/EBPα* and *PPARγ*) was analyzed. The differentiation of 3T3-L1 cells into adipocytes was induced by incubating in MDI induction medium for 2 days followed by incubation in differentiation medium for an additional 6 days. After MDI-induced adipocyte differentiation of 3T3-L1 cells, adipogenic transcriptional factors were analyzed. The transcriptional factors C/EBPα and PPARγ were inhibited by **8n** and **8o** in terms of both protein (Fig. 2C,D) and RNA (Fig. 2E,F) levels. In addition, the mRNA expression of the genes downstream of PPARγ including adiponectin and fatty acid synthase markers (FAS) were strongly reduced by treatment with **8o**. The results indicated that isoquinolinoquinazolinones may suppress adipogenesis by affecting the PPARγ pathway.

**Figure 3:**
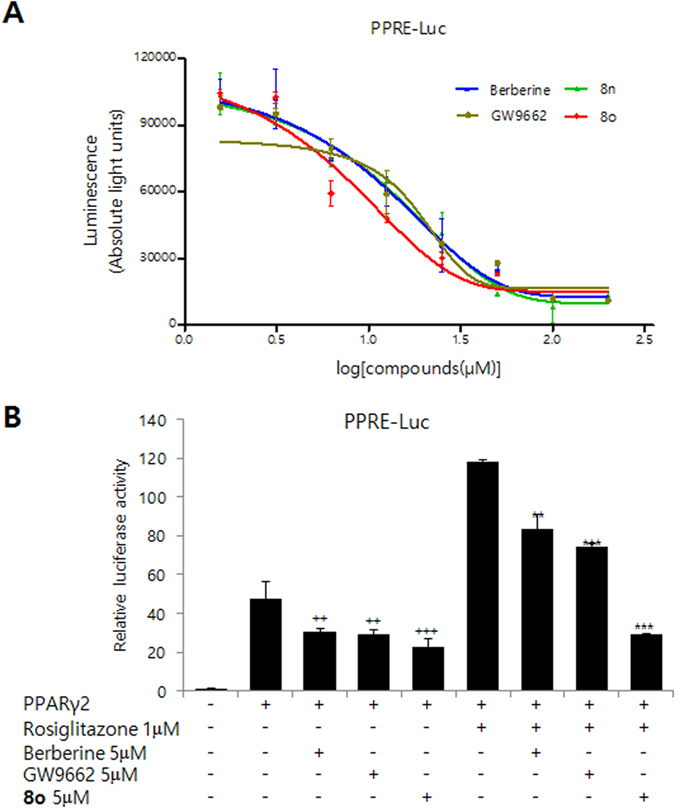
Effect of 8o on PPARγ-mediated transcriptional activity as evidenced by PPRE promoter activity. (**A**) Inhibitory effect of **8o** on PPARγ transcriptional activity. The transcription activity was determined in 3T3-L1 cells transiently co-transfected with PPRE-driven luciferase reporter gene (PPRE-Luc) and PPARγ2 expression vector. The results are presented as luminescence value after treatment with berberine, **8n**, **8o**, and GW9662. (**B**) Inhibitory activities of **8o** on rosiglitazone mediated PPARγ transcription activities. The transcriptional activity was determined in 3T3-L1 cells transiently co-transfected with PPRE-driven luciferase reporter gene (PPRE-Luc) and PPARγ2 expression vector. After 24 h of transfection, the cells were cultured with indicated combination for 12 h. Luciferase activity was measured after 36 h. Isoquinolinoquinazolinone can competitively inhibit PPARγ2-induced transcriptional activity in the presence of rosiglitazone. Data show mean ± SD of at least three experiments. ^++^P < 0.01, ^+++^P < 0.001 as compared to cells transfected with PPARγ alone. ^**^P < 0.01, ^***^P < 0.001 as compared to cells transfected with PPARγ and treated with rosiglitazone.

To analyze whether isoquinolinoquinazolinones inhibit the transactivation of PPARγ, 3T3-L1 cells were cotransfected with full-length PPARγ2 expression vector with a peroxisome proliferator response element (PPRE)-driven luciferase reporter gene (PPRE-Luc) that has been reported to respond to PPARγ^23^,^24^. The effectiveness of PPARγ2 transfection was inhibited by every compound tested (berberine, GW9662, **8n** and **8o**) in a dose-dependent manner. Titration curves were generated using Graph Pad Prism^®^, and the IC_50_ value of each compound with respect to PPARγ2-induced transcriptional activity was determined. The IC_50_ of **8o** was 2.43 μM, while the IC_50_ values of **8n**, berberine, and GW9662 were 5.02 μM, 6.63 μM, and 4.47 μM, respectively. Compound **8o** showed the greatest inhibitory effect (lowest IC_50_ value) on PPARγ2 transcriptional activity (Fig. 3A). To examine isoquinolinoquinazolinone-mediated inhibition of ligand-induced PPARγ activity, the PPARγ agonist rosiglitazone was used. Luciferase expression was increased by up to 3-fold by rosiglitazone treatment. Treatment with a 5 μM dose of **8o** either alone or with rosiglitazone stimulation resulted in the inhibition of PPARγ2 transfection by 82% and 88%, respectively. This was greater than berberine (33% and 40%) and GW9662 (46% and 48%) (Fig. 3B). The results showed that isoquinolinoquinazolinones were more potent inhibitors of PPARγ2-induced transcriptional activity than GW9662 in both the absence and presence of the PPARγ agonist rosiglitazone.

**Figure 4:**
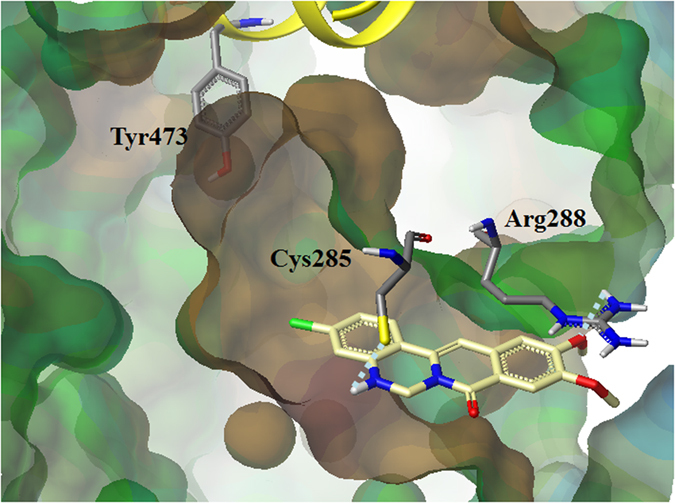
Molecular docking mode of 8o in PPARγ (PDB: 3E00).

A molecular modeling study was conducted to examine the hypothetical binding modes of PPARγ and isoquinolinoquinazolinones including **8o** that exhibited potent inhibitory activities. The surflex-Dock program in Sybyl-X 2.1.1 was used to dock isoquinolinoquinazolinones into the ligand binding site of PPARγ following the removal of the ligand from the 3D crystal structure of PPARγ-GW9662-RXRα-retinoic acid-NCoA-2-DNA complex (PDB: 3E00). According to the docking model, isoquinolinoquinazolinone **8o** occupied the hydrophobic region of PPARγ-LBP (Fig. 4). The amino group in ring B and methoxyl group in ring D formed hydrogen bonds with Cys285 and Arg288, respectively. Moreover, isoquinolinoquinazolinone **8o** was located far away from H12. Furthermore, docking models showed that the isoquinolinoquinazolinones like **8l**, which lacked significant inhibitory effect on adipocyte differentiation (39.1%), despite of subtle structural difference (absence of 10-OCH_3_) than the potent counterpart like **8o** (inhibitory effect on adipocyte differentiation: 82.9%) was basically due to unfavorable orientation of the compound in the LBP (Fig. S2).

A surface plasmon resonance (SPR) analysis demonstrated that **8o** binds to PPARγ2-LBD (Fig. S1). A Reichert SR7500 (Reichert, Depew, NY) biosensor was used to measure the binding affinity of rosiglitazone, GW9662, and **8o** with PPARγ2-LBD. PPARγ2 protein was immobilized on the sensor chip, and the response (in resonance unit; RU) as a result of binding was continuously recorded and presented graphically as a function of time^25^. The association rate (k_a_ also known as on rate (k_on_)), dissociation rate (k_d_ also known as off rate (k_off_)), and equilibrium dissociation constant (K_D_) were calculated using the Scrubber 2 program (Table 2). Rosiglitazone binds to PPARγ2 quickly (k_a_: 24 M^−1^s^−1^) and dissociates easily (k_d_: 0.0867 s^−1^) (Fig. S1A). On the other hand, GW9662 binds slowly to PPARγ2 (k_a_: 15.09 M^−1^s^−1^) but hardly dissociates (k_d_: 0.000059 s^−1^) from the protein confirming the irreversible covalent linkage of the ligand with PPARγ (Fig. S1B). Compound **8o** binds and dissociates from PPARγ with intermediate rates (k_a_: 24 M^−1^s^−1^; k_d_: 146.25 s^−1^) (Fig. S1C). The binding affinity of **8o** to PPARγ is 4-folds more than rosiglitazone (K_D_: 146.25 μM (**8o**), 581.88 μM (rosiglitazone)) but is 37-folds less than GW9662 (K_D_: 3.91 μM). The SPR result correlates well with the competitive assay of **8o** and rosiglitazone.

**Figure 5:**
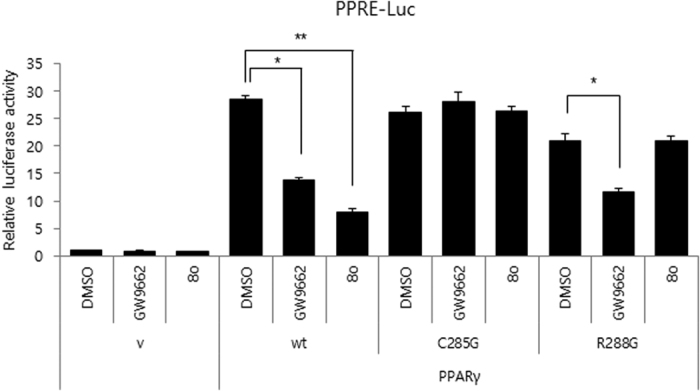
Mutational study. Residues Cys285 and Arg288 of PPARγ are important for **8o** function. The transcriptional activity was determined in 3T3-L1 cells transiently co-transfected with PPRE-driven luciferase reporter gene (PPRE-Luc) and PPARγ2 (WT) or (C285G, R288G) expression vector. After 24 h of transfection, the cells were cultured with 5 μM GW9662 and 5 μM **8o** respectively for 12 h. Luciferase activity was measured after 36 h. Error bars mean ± SD of at least three experiments. ^*^P < 0.05, ^**^P < 0.01.

To investigate the contribution site on the PPARγ2 to the interaction with **8o**, we generated mutant forms of the PPARγ2 (Fig. 5). In the docking results, **8o** showed the interaction with Cys285 and Arg288. Thus, we tested Cys-to-Gly (Cys285), Arg-to-Gly (Arg288) substitution mutants of PPARγ2. Interestingly, the substitution of Cys285 to Gly abolished the inhibitory effect of both GW9662 and **8o** on PPARγ2 activity. However, the substitution of Arg288 to Gly abolished the inhibitory effect of **8o** not in GW9662. The results indicated **8o** has interaction with both Cys285 and Arg288, which are in agreement with the docking study.

## Conclusion

Based on the PPARγ antagonist behavior of berberine and the fact that the LBD of PPARγ contains a coactivator binding site, we designed a series of isoquinolinoquinazolinones. This novel class of PPARγ antagonists was synthesized in two steps and inhibited 3T3-L1 adipocyte differentiation by inhibiting transcription factor PPARγ. Compound **8o** more effectively suppressed PPARγ transactivation than the existing PPARγ antagonist GW9662. The potent inhibitory effect of the isoquinolinoquinazolinones was well explained by their docking mode in the LBP of PPARγ. A molecular modeling study showed that, as expected, it was the presence of a quinazolinone -NH in ring B that enabled the isoquinolinoquinazolinones to have higher PPARγ inhibitory activities than GW9662. All of the biological assays, the SPR results and mutation study indicated that we have successfully designed and synthesized a new series of potent PPARγ antagonists. SAR data and docking models can provide helpful guidance in designing PPARγ antagonists. All of these findings suggest that isoquinolinoquinazolinones might exert multiple therapeutic effects and are potential treatments for obesity, type 2 diabetes, hyperlipidemia, and other metabolic syndromes.

## Experimental Procedures

### General Information and Instrumentation

Melting points were determined using the capillary method with a MEL-TEMP^®^ capillary melting point apparatus and were uncorrected. IR spectra were obtained on a JASCO FT/IR 300E Fourier transform infrared spectrometer using KBr pellets. ^1^H NMR and ^13^C NMR spectra were recorded with Varian Unity Plus 300 MHz and Varian Unity Inova 500 MHz spectrometers at the Korea Basic Science Institute. Chemical shifts are reported in parts per million (ppm) downfield relative to TMS (*δ* = 0). The coupling constants *J* are given in Hertz. The data are reported in the following order: chemical shift, multiplicity, coupling constant, and number of protons. Multiplicity of proton signals is reported as s: singlet, d: doublet, t: triplet, q: quartet, m: multiplet, dd: double doublet, td: triplet of doublets, bs: broad single. Mass spectra were obtained on a Shimadzu LCMS-2010 EV liquid chromatograph mass spectrometer using the electron spray ionization (ESI) method. Elemental analyses were performed using a Thermo Fischer Flash 2000 elemental analyzer and all measured values are within ± 0.3% of the theoretical values. Column chromatography was performed with Merck silica gel 60 (70–230 mesh). TLC was performed using plates coated with silica gel 60 F254 (Merck). Chemical reagents were purchased from Sigma-Aldrich and Tokyo Chemical Industry Co., Ltd. and were used without further purification. Solvents were distilled prior to use; THF was distilled from sodium/benzophenone. All reactions were conducted under a nitrogen atmosphere in oven-dried glassware with magnetic stirring. The specifications of HPLC analysis are as follows: column, ACE C18-HL, 250 × 2.1 mm, flow rate, 0.2 mL/min; wavelength, 254 nm; mobile phase; acetonitrile:water (9:1, v/v). The purity of compounds was established by integration of the areas of all peaks detected and is reported for each final compound. All compounds tested in the biological assay showed more than 95% purity.

### Synthesis of isoquinolinoquinazolinones

3-(2-Aminophenyl)isoquinolin-1(*2H*)-one (**7a**). A 100-mL oven-dried, three-necked flask was sealed with septa and evacuated/backfilled with nitrogen gas (N_2_) three times before starting the reaction. A solution of *N,N*-diethyl-2-methyl-benzamide **5a** (13.1 g, 68.8 mmol) and 2-aminobenzonitrile **6a** (8.12 g, 68.8 mmol) in dry THF (30 mL) was added drop wise to a solution of n-BuLi (2.5 M solution in hexane; 82 mL) in dry THF (20 mL) at −60 °C, and the reaction mixture was stirred at −78 °C overnight. The reaction mixture was quenched with saturated NH_4_Cl solution (100 mL). The mixture was added to water (200 mL), extracted with CH_2_Cl_2_, and washed with water and brine. The organic phase was dried over sodium sulfate and concentrated *in vacuo*. The resulting oil was purified by column chromatography eluting with *n*-hexane:ethyl acetate (2:1) to afford compound **7a** as a yellow solid (9.07 g, 56%). Mp: 236.5–238.1 °C. ^1^H NMR (300 MHz, DMSO-*d*_6_) *δ*: 8.18 (d, *J* = 6.6 Hz, 1H), 7.71–7.62 (m, 2H), 7.48–7.43 (m, 1H), 7.13–7.08 (m, 2H), 6.77–6.74 (m, 1H), 6.62 (td, *J* = 7.5, 1.2 Hz, 1H), 6.60 (s, 1H), 5.14 (bs, 2H). ^13^C NMR (125 MHz, DMSO-*d*_6_) *δ*: 162.7, 146.1, 139.2, 138.1, 132.2, 129.7, 129.6, 126.3, 124.9, 119.1, 115.9, 115.4, 104.5. MS (ESI) *m/z* = 237 (M + H)^+^. Anal. Calcd. for C_15_H_12_N_2_O • 0.05 C_4_H_10_O_2_: C, 75.82; H, 5.23; N, 11.86. Found: C, 75.52; H, 5.19; N, 11.63.

3-(2-Amino-5-chlorophenyl)-6,7-dimethoxyisoquinolin-1(*2H*)-one (**7n**). The procedure used to prepare compound **7a** was carried out using compound **5g** (800 mg, 3.18 mmol), **6b** (485 mg, 3.18 mmol), and *n*-BuLi (2.5 M solution in hexane, 4 mL) to afford compound **7n** as ivory solid (266 mg, 25%). Mp: 272.7–274.1 °C. ^1^H NMR (300 MHz, DMSO-d_6_) *δ*: 11.07 (bs, 1H), 7.56 (s, 1H), 7.17–7.14 (m, 1H), 7.13–7.10 (m, 2H), 6.77–6.74 (m, 1H), 6.53 (s, 1H), 5.29 (bs, 2H), 3.88 (s, 3H), 3.87 (s, 3H). MS (ESI) *m/z* = 331 (M + H)^+^. Anal. Calcd. for C_17_H_15_ClN_2_O_3_ • 0.35 CH_2_Cl_2_ • 0.35 C_3_H_7_NO: C, 57.24; H, 4.74; N, 8.53. Found: C, 57.43; H, 4.74; N, 8.70.

3-(2-Amino-4-chlorophenyl)-6,7-dimethoxyisoquinolin-1(*2H*)-one (**7o**). The procedure used to prepare compound **7a** was carried out using compound **5g** (800 mg, 3.18 mmol), **6c** (485 mg, 3.18 mmol), and *n*-BuLi (2.5 M solution in hexane, 4 mL) to afford compound **7o** as an ivory solid (118 mg, 11%). Mp: 250.6–251.5 °C. ^1^H NMR (300 MHz, DMSO-d_6_) *δ*: 11.06 (bs, 1H), 7.56 (s, 1H), 7.17 (s, 1H), 7.08 (d, *J* = 6.0 Hz, 1H), 6.78 (d, *J* = 3.0 Hz, 1H) 6.60 (dd, *J* = 9.0, 3.0 Hz, 1H), 6.49 (s, 1H), 5.43 (bs, 2H), 3.87 (s, 3H), 3.86 (s, 3H). Anal. MS (ESI) *m/z* = 331 (M + H)^+^. Calcd for C_17_H_15_ClN_2_O_3_ • 0.15 CH_2_Cl_2_ • 0.15 C_3_H_7_NO: C, 59.64; H, 4.65; N, 8.32. Found: C, 59.68; H, 4.78; N, 8.33.

*5H*-Isoquinolino[2,3-c]quinazolin-8(*6H*)-one (**8a**). A suspension of compound **7a** (4 g, 16.9 mmol), and K_2_CO_3_ (5.85 g, 42.3 mmol) in DMF (10 mL) was stirred for 30 min at room temperature, then treated with diiodomethane (18.1 g, 67.7 mmol). After refluxing for 4.0 h, the solvent was evaporated, and the crude product was dissolved in dichloromethane, washed with brine, dried over sodium sulfate, and concentrated *in vacuo*. The resulting mixture was purified by chromatography eluting with *n*-hexane:ethyl acetate (4:1) to afford **8a** as a yellow solid (976 mg, 23%). Mp: 204.3–206.3 °C. IR (cm^−1^): 3289, 1588. ^1^H NMR (300 MHz, DMSO-d_6_) *δ*: 8.20 (d, *J* = 7.8 Hz, 1H), 7.86 (d, *J* = 7.2 Hz, 1H), 7.71–7.69 (m, 2H), 7.48–7.41 (m, 2H), 7.28–7.22 (m, 1H), 7.17 (s, 1H), 6.92–6.89 (m, 1H), 6.83 (s, 1H), 5.15 (d, *J* = 2.1 Hz, 2H). MS (ESI) *m/z* = 249 (M + H)^+^. Anal. Calcd. for C_15_H_12_N_2_O • 0.05 H_2_O • 0.15 C_4_H_8_O_2_: C, 76.35; H, 5.04; N, 10.86. Found: C, 76.17; H, 4.86; N, 10.69. HPLC: t_r_ 2.00 min, purity 98.8%.

2-Chloro-10,11-dimethoxy-*5H*-isoquinolino[2,3-c]quinazolin-8(*6H*)-one (**8n**). The procedure used to prepare compound **8a** was carried out using compound **7n** (200 mg, 0.60 mmol), K_2_CO_3_ (209 mg, 1.51 mmol), and diiodomethane (643 mg, 2.40 mmol) to afford compound **8n** as a yellow solid (98 mg, 47%). Mp: 193.5–194.6 °C. IR (cm^−1^): 3286, 1571. ^1^H NMR (300 MHz, DMSO-d_6_) *δ*: 7.83 (d, *J* = 3.0 Hz, 1H), 7.56 (s, 1H), 7.26–7.18 (m, 3H), 6.94 (bs, 1H), 6.90 (d, *J* = 9.0 Hz, 1H), 5.12 (d, *J* = 3.0 Hz, 2H), 3.90 (s, 3H), 3.87 (s, 3H). ^13^C NMR (125 MHz, DMSO-d_6_) *δ*: 158.8, 153.3, 148.8, 144.0, 132.7, 132.0, 129.5, 123.5, 123.1, 119.2, 118.0, 117.5, 107.0, 106.9, 100.4, 55.6, 55.5. MS (ESI) *m/z* = 343 (M + H)^+^. Anal. Calcd. for C_18_H_15_ClN_2_O_3_ • 0.1 H_2_O • 0.05 C_4_H_8_O_2_ • 0.8 CH_2_Cl_2_: C, 54.73; H, 4.16; N, 6.72. Found: C, 54.84; H, 4.05; N, 6.61. HPLC: t_r_ 1.99 min, purity 98.3%.

3-Chloro-10,11-dimethoxy-*5H*-isoquinolino[2,3-c]quinazolin-8(*6H*)-one (**8o**). The procedure used to prepare compound **8a** was carried out using compound **7o** (100 mg, 0.37 mmol), K_2_CO_3_ (104 mg, 0.75 mmol), and diiodomethane (321 mg, 1.20 mmol) to afford compound **8o** as a yellow solid (58 mg, 56%). Mp: 228.7–230.5 °C. IR (cm^−1^): 3305, 1591. ^1^H NMR (300 MHz, DMSO-d_6_) *δ*: 7.80 (d, *J* = 3.0 Hz, 1H), 7.56 (s, 1H), 7.18 (s, 1H), 7.12 (s, 1H), 7.01 (s, 1H), 6.94–6.89 (m, 2H), 5.14 (d, *J* = 3.0 Hz, 2H), 3.90 (s, 3H), 3.87 (s, 3H). ^13^C NMR (125 MHz, DMSO-d_6_) *δ*: 158.8, 153.3, 148.6, 146.4, 134.3, 133.2, 132.0, 125.9, 119.2, 116.5, 114.9, 107.0, 106.8, 99.8, 55.7, 55.5, 52.0. MS (ESI) *m/z* = 343 (M + H)^+^. Anal. Calcd. for C_18_H_15_ClN_2_O_3_ • 0.1 C_4_H_8_O_2_ • 0.1 CH_2_Cl_2_: C, 61.71; H, 4.48; N, 7.78. Found: C, 61.62; H, 4.31; N, 7.60. HPLC: t_r_ 1.93 min, purity 99.0%.

6,8-Dihydro-*5H*-isoquinolino[2,3-c]quinazoline (**9**). Lithium aluminum hydride (550 mg, 14.5 mmol) was added portion-wise to a stirred solution of **8o** (400 mg, 1.61 mmol) in dry THF (20 mL) in a stream of nitrogen at 0 °C and stirring was continued for 1 h at room temperature. Thereafter, the reaction mixture was diluted with water and filtered. The filtrate was dried and concentrated *in vacuo*. The residue was purified by column chromatography eluting with *n*-hexane:ethyl acetate (5:1) to afford compound **9** as an ivory solid (110 mg, 30%). ^1^H NMR (300 MHz, CDCl_3_) *δ*: 8.66 (bs, 1H), 8.40 (d, *J* = 7.8 Hz, 1H), 7.70–7.65 (m, 1H), 7.57–7.47 (m, 2H), 7.38–7.32 (m, 1H), 7.27–7.24 (m, 1H), 6.82 (td, *J* = 7.5, 0.6 Hz, 1H), 6.75 (d, *J* = 8.1 Hz, 1H), 6.61 (s, 1H), 4.15 (s, 1H), 2.84 (d, J = 4.8 Hz, 3H).). ^13^C NMR (125 MHz, DMSO-d_6_) *δ*: 162, 153, 149, 147, 136, 135, 133, 130, 117, 110, 107, 106, 56, 30. MS (ESI) *m/z* = 329 (M + H)^+^. HPLC: t_r_ 1.92 min, purity 98.9%.

### Plasmids

For plasmids expressing PPARγ2, full length PPARγ2 (mouse, 1518 bp: NCBI Reference Sequence: NP_035276.2) was subcloned into CMV promoter-derived mammalian expression vector (pCS4). Plasmids for C285G, R288G mutant PPARγ2 were generated by PCR-based site-directed mutagenesis and also subcloned into CMV promoter-derived mammalian expression vector (pCS4). Sequence of mutants was confirmed by Xenotech biology (Daejeon, Korea).

### Cell culture and differentiation conditions

The mouse preadipocyte cell line 3T3-L1 was maintained at 37 °C in humidified air with 5% CO_2_. 3T3-L1 cells were cultured in Dulbecco’s modified Eagle medium (DMEM; Life Technologies) supplemented with 10% bovine serum (BS; Gibco Invitrogen, Carlsbad, California, USA) as growth medium. For adipocyte differentiation, cells were cultured for 2 days to full confluence in a 24-well plate and the growth medium was then replaced (day 0) with DMEM supplemented with 10% fetal bovine serum (FBS, Gibco Invitrogen, Carlsbad, California, USA), 5 μg/mL insulin (Sigma, St. Louis, Missouri, USA), 0.5 mM 3-isobutyl-1-methylxanthine (Sigma), and 1 μM dexamethasone (Sigma). After 48 h, the differentiation medium was replaced (day 2) with DMEM + 10% FBS containing 5 μg/mL insulin, and the cells were allowed to accumulate lipid droplets until experimental use.

### Luciferase reporter assays

3T3-L1 cells were transfected for indicated combinations of expression plasmids along with a luciferase reporter plasmid (PPRE-Luc). PPRE-Luc contains the consensus PPAR responsive element (PPRE). pCMV-β-gal was co-transfected for normalization of transfection efficiency. 24 h after transfection, cells were treated with indicated compounds for 12 h. Luciferase activities were measured using a luciferase reporter assay kit (Promega, Madison, WI, USA). Experiments were performed in triplicate and repeated at least three times.

### Oil Red O Staining

The accumulation of lipids signifying the formation of adipocytes was observed by staining the differentiated cells with Oil Red O. Following differentiation, cells were washed twice with phosphate-buffered saline (PBS), fixed with 10% formalin for 60 min. Oil Red O stock solution (0.5%) was prepared in isopropanol and filtered in cellulose nitrate filters. Cells were stained with Oil Red O diluted 6:4 in water for 1 h at room temperature. Excess Oil Red O dye was washed off twice with distilled water and then dried. The stained lipid droplets within cells were visualized using an optical microscope and photographed with a digital camera at 100× magnification.

### RT-PCR analyses

Total cellular RNA was prepared using TRIzol reagent (Life Technologies) according to the manufacturer’s instructions. Random-primed cDNAs were synthesized from 1 μg of total RNA using Super-Script III First-Strand Synthesis System (Life Technologies). The following conditions were used for PCR: initial denaturation at 94 °C for 1 min; 28–30 cycles of denaturation at 94 °C for 30 s, annealing at a temperature optimized for each primer pair for 30 s, extension at 72 °C for 30 s; final extension at 72 °C for 5 min. The following PCR primers were used: *C/EBPα* forward 5′-TGC TGG AGT TGA CCA GTG AC-3′ and reverse 5′-AAA CCA TCC TCT GGG TCT CC-3′; PPARγ forward 5′-ATC AGC TCT GTG GAC CTC TC-3′ and reverse 5′-ACC TGA TGG CAT TGT GAG AC-3′; Adiponectin forward 5′-CAT CCC AGG ACA TCC TGG CCA CAA TG-3′ and reverse 5′-GGC CCT TCA GCT CCT GTC ATT CCA AC-3′; FAS forward 5′-GCT ATG CAG ATG GCT GTC TCT CCC AG-3′ and reverse 5′-GCA GCG CTG TTT ACA TTC CTC CCA GG-3′; GAPDH forward 5′-ACC ACA GTC CAT GCC ATC AC-3′ and reverse 5′-TCC ACC ACC CTG TTG CTG TA-3′. RNA levels were quantified by image software, Multi Gauge, V3.0 (FUJIFILM).

### Immunoblotting (IB)

At the end of differentiation, 3T3-L1 cells were washed with PBS and lysed in a lysis buffer (1% NP-40, 25 mM HEPES (pH 7.5), 10% glycerol, 150 mM NaCl, 25 mM NaF, 0.25% sodium deoxycholate, 1 mM EDTA, 1 mM Na_3_VO_4_, 10 μg/mL aprotinin, 10 μg/mL leupeptin, and 250 μM phenylmethanesulfonyl fluoride). For immunoblotting (IB), Aliquots from the cell lysates containing 30 μg of protein were heated at 95 °C for 5 min and were then separated using SDS-PAGE, and proteins were transferred to PVDF membrane. Blots were blocked for 30 min with Tris-buffered saline (50 mM Tris.HCl, pH 7.4, 150 mM NaCl)-0.05% Tween 20 (TBS-T) supplemented with 5% skim milk. The membranes were subsequently incubated with various primary antibodies; PPARγ (#MAB3872, 1:1000, Chemicon International), C/EBPα (#04-1104, 1:1000, Upstate Biotechnology), and α-tubulin (#B-5-1-2, 1:5000, Sigma-Aldrich). Horseradish peroxidase-labeled secondary antibodies were detected and visualized using chemiluminescent western blotting reagent (Millipore). Protein expression levels were quantified by image software, Multi Gauge, V3.0 (FUJIFILM).

### Molecular docking

The docking study was performed in Sybyl-X 2.1.1 (winnt_os5x) using the Surflex Dock program. The structure of PPARγ-GW9662-RXRα-retinoic acid-NCoA-2-DNA complex was downloaded from the Protein Data Bank (PDB: 3E00). Structures of RXRα, retinoic acid, NCoA-2, and DNA were removed. The ligand (GW9662) was extracted. Hydrogens were added and minimization was performed using the MMFF94s force field with MMFF94 charges, by using a conjugate gradient method, distance dependent dielectric constant and converging to 0.01 kcal/mol Å. Protomol, an idealized representation of a ligand that makes every potential interaction with the binding site, was generated on the basis of ligand mode. Oxaprotoberberines and isoquinolinoquinazolines were constructed in Sybyl; energy was minimized with MMFF94s force field and MMFF94 charges and stored in a Sybyl database. The compounds in the Sybyl database were docked into the binding site by Surflex Dock on the basis of the protomol constructed earlier.

### Expression and purification of mouse PPARγ2 ligand binding domain (PPARγ2–LBD) protein

The recombinant ligand-binding domains (LBDs) of mouse PPARγ2 (residues 230–505) were expressed as N-terminal GST-tagged proteins using the pGEX4T3 vector. pGXE4T3-mPPARγ2-LBD plasmid was transfected into BL21(DE3) competent cells and the cells were grown in LB medium containing 50 μg/mL ampicillin at 37 °C to an O. D. 600 of 0.6–0.8. The expression of PPARγ2-LBD was induced by the addition of 0.2 mM isopropyl β-d-thiogalactoside (IPTG). After treatment for 24 h at 22 °C, the cells were harvested and disrupted using sonication buffer [25 mM HEPES (pH 7.5), 150 mM NaCl, 1% NP-40, 0.25% sodium deoxycholate, 10% glycerol, 25 mM NaF, 1 mM EDTA, 1 mM Na_3_VO_4_, 250 μM PMSF, 10 μg/mL leupeptin, and 10 μg/ml aprotinin]. The supernatant was applied to glutathione-Sepharose beads for 24 h at 4 °C. The bound GST fusion proteins were washed with the sonication buffer and eluted by elution buffer [25 mM glutathione, 50 mM Tris pH 8.8, 200 mM NaCl].

### Surface Plasmon Resonance analysis

Analysis of the interaction between immobilized PPARγ2-LBD and rosiglitazone, GW9662 and **8o** was performed using Reichert SR7500 Surface Plasmon Resonance dual channel instrument (Reichert, Depew, NY). Immobilization of the protein on the hydrophilic carboxymethylated dextran matrix of the sensor chip (Reichert) was carried out using a standard primary amine coupling reaction. Free carboxyl groups on the surface were modified by injecting a mixture of 0.1 M 1-ethyl-3-(3-dimethylaminopropyl) carbodiimide hydrochloride and 0.05 M *N*-hydroxysuccinimide at a flow rate of 20 μL/min to generate a reactive succinimide ester surface. Baseline equilibration was achieved by flushing the chip with PBS buffer for 1–2 h. All of the SPR data was collected at 25 °C with PBS as running buffer at a constant flow of 30 μL/min. All the sensorgrams were processed by using automatic correction for nonspecific bulk refractive index effects. All the kinetic analyses of the binding to PPARγ2-LBD were calculated using the Scrubber2 program (Bio-Logic Software).

### Statistical analysis

The results are expressed as the means ± standard error of the means (SEM). Data were analyzed using Student’s t-test, with p < 0.05 indicating significance. All experiments were performed in triplicates and were repeated at least three times. Results of representative experiments are shown.

## Additional Information

**How to cite this article**: Jin, Y. *et al*. Discovery of Isoquinolinoquinazolinones as a Novel Class of Potent PPARγ Antagonists with Anti-adipogenic Effects. *Sci. Rep*. **6**, 34661; doi: 10.1038/srep34661 (2016).

## Supplementary Material

Supplementary Information

## Figures and Tables

**Table 1:**
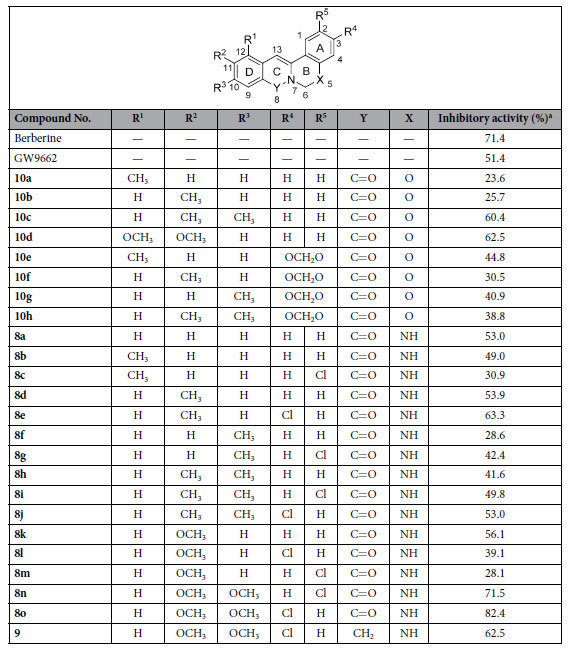
Inhibitory activity of 5-oxaprotoberberines **10** and isoquinolinoquinazolinones **8** on adipocyte differentiation.

^a^Relative absorbance data from Oil Red O staining assay at 25 μM.

**Table 2:** The kinetic constants of rosiglitazone, GW9662, and **8o** binding to PPARγ2-LBD.

Analyte	k_a_ (M^−1^s^−1^)	k_d_ (s^−1^)	K_D_ (μM)
Rosiglitazone	149	0.0867	581.88
GW9662	15.09	0.000059	3.91
8o	24	0.00351	146.25

k_a_: association rate constant, k_d_: dissociation rate constant and K_D_: equilibrium dissociation constant.
