# Supplementary material for: Discovery of Isoquinolinoquinazolinones as a Novel Class of Potent PPARγ Antagonists with Anti-adipogenic Effects
Source: Sci Rep. 2016 Oct 3;6:34661. doi: 10.1038/srep34661 (PMC5046141; doi:10.1038/srep34661)
Supplement: Supplementary Information [file srep34661-s1.pdf]

Supplementary Information

Discovery of Isoquinolinoquinazolinones as a Novel Class of Potent PPARγ Antagonists with Anti-adipogenic Effects

Yifeng Jin,[†] Younho Han,[†] Daulat Bikram Khadka, Chao Zhao, Kwang Youl Lee,* Won-Jea Cho*

College of Pharmacy and Research Institute of Drug Development, Chonnam National University, Gwangju 61186, Republic of Korea

*To whom correspondence should be addressed:

Won-Jea Cho, Ph.D., phone: +82-62-530-2933. Fax: +82-62-530-2911. E-mail: wjcho@chonnam.ac.kr.

Kwang Youl Lee, Ph.D., phone: +82-62-530-2939. Fax: +82-62-530-2911. E-mail: kwanglee@chonnam.ac.kr.

[†]These authors contributed equally to this work.

Table of Contents

Scheme S1........S3

Scheme S2........S3

Figure S1........S4

Figure S2........S5

Figure S3........S6

Figure S4........S6

Figure S5........S7

Figure S6........S7

Supplemental Experimental procedures and characterization data of the synthesized compounds

Synthesis of isoquinolinoquinazolinones S8

^{1}H-NMR and ^{13}C-NMR spectra of **8n**........S19

^{1}H-NMR and ^{13}C-NMR spectra of **8o**........S20

Scheme S1. Synthesis of isoquinolinoquinazolinones 8[a]

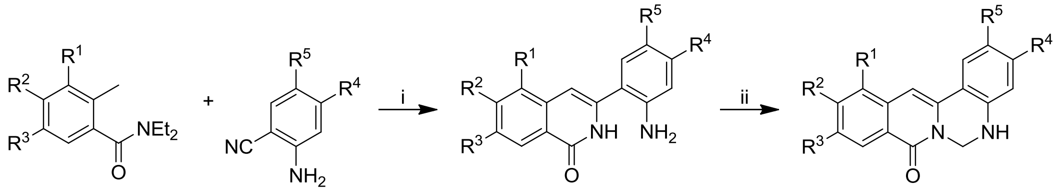

5 **6** **7a-o** **8a-o**

	R^1	R^2	R^3		R^4	R^5
**5a**	H	H	H	**6a**	H	H
**5b**	CH_3	H	H	**6b**	H	Cl
**5c**	H	CH_3	H	**6c**	Cl	H
**5d**	H	H	CH_3			
**5e**	H	CH_3	CH_3			
**5f**	H	OCH_3	H			
**5g**	H	OCH_3	OCH_3			

[a]Reagents and conditions: (i) *n*-BuLi, dry THF, −78 °C; (ii) CH_2I_2, K_2CO_3, DMF, 100 °C.

Scheme S2. Synthesis of 5,8-dihydro-6*H*-isoquino[*2,3-c*]quinazoline 9.[a]

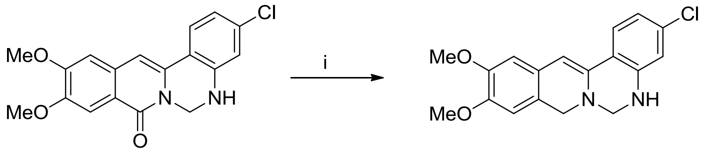

8o **9**

[a]Reagents and conditions: (i) $LiAlH_4$, dry THF, MeOH.

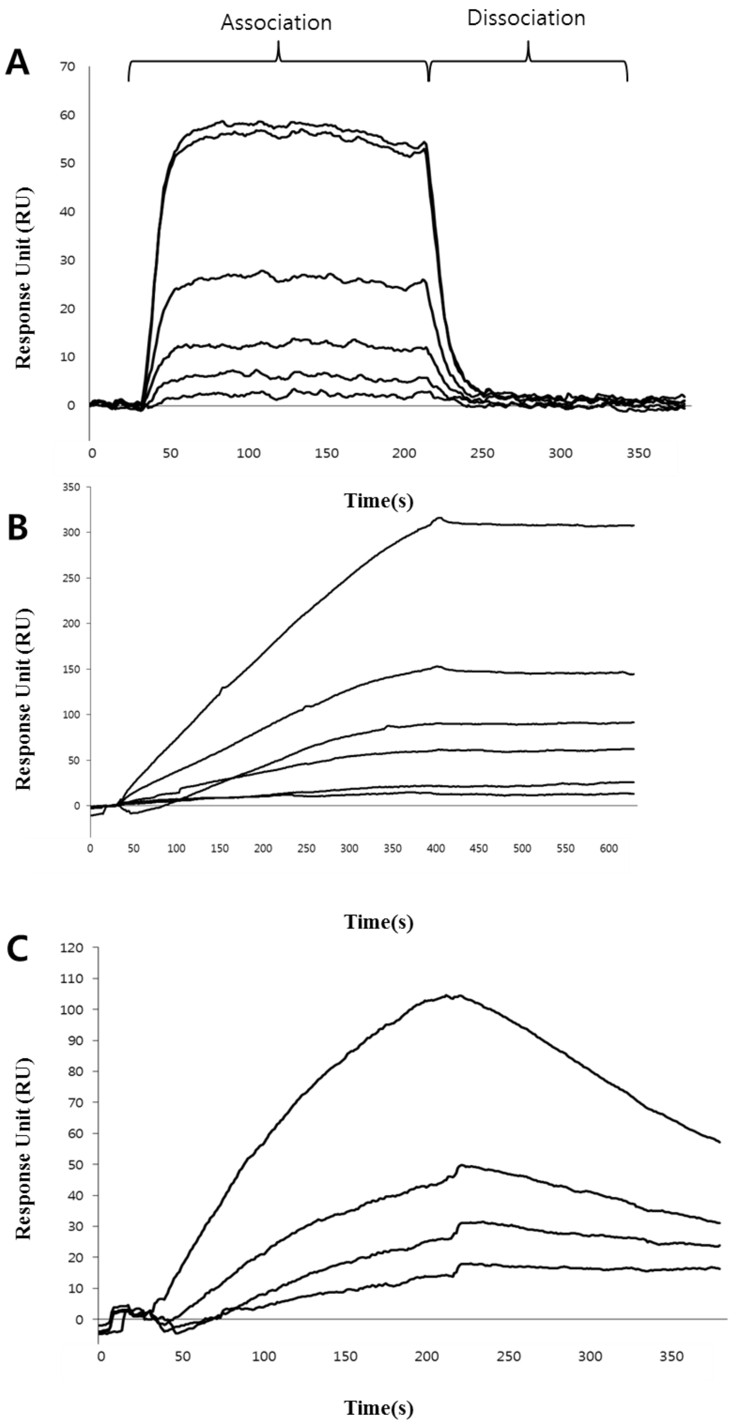

Figure S1. SPR analysis. Representative sensorgrams were obtained by immobilizing PPAR protein on the sensor chip and treating with (**A**) rosiglitazone at concentrations of 2.5, 5, 10, 20, 40, and 80 μM; (**B**) GW9662 at concentrations of 3.125, 6.25, 12.5, 25, 50, and 100 μM; (**C**) **8o** at concentrations of 7.5, 15, 30, and 60 μM.

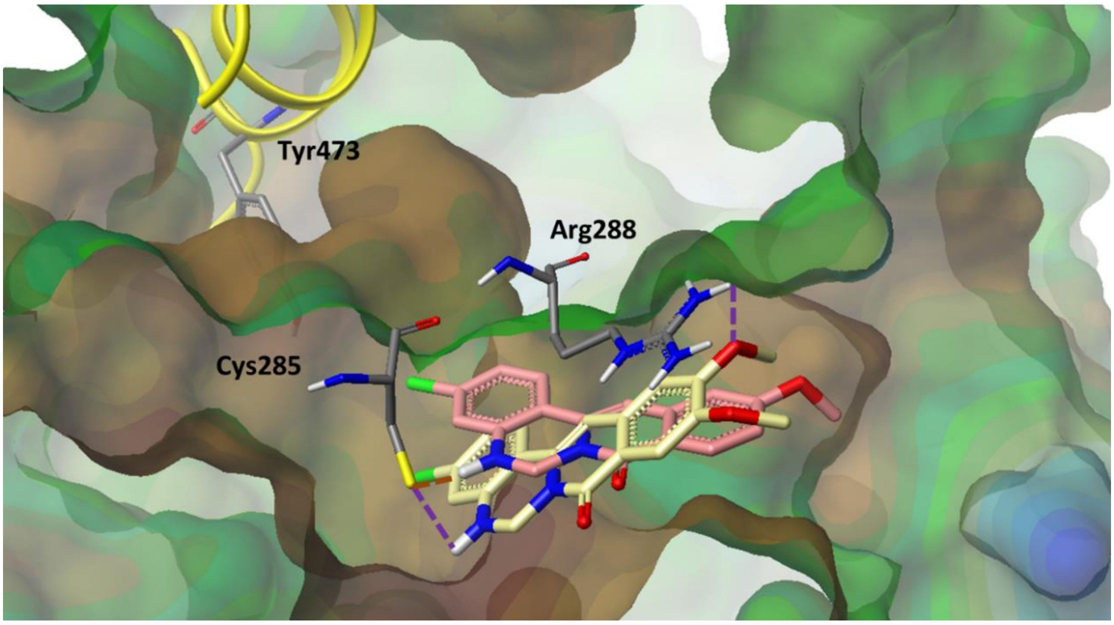

Figure S2. Molecular docking mode of 8l (pink), 8o (yellow) and PPARγ (PDB: 3E00). H-bonds are shown as brown (for **8l**) and purple (for **8o**) discontinuous lines. Compound **8l** lacks H-bond interaction with Arg288.

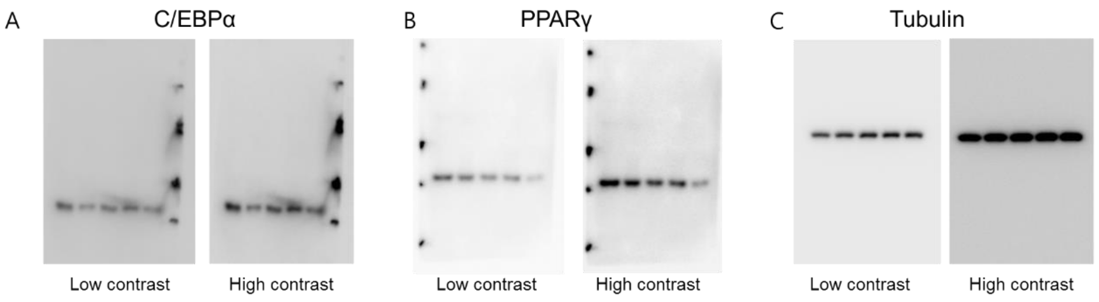

Figure S3. Full-length membrane blots corresponding to Fig 2D (C/EBPα, PPARγ, and Tubulin).

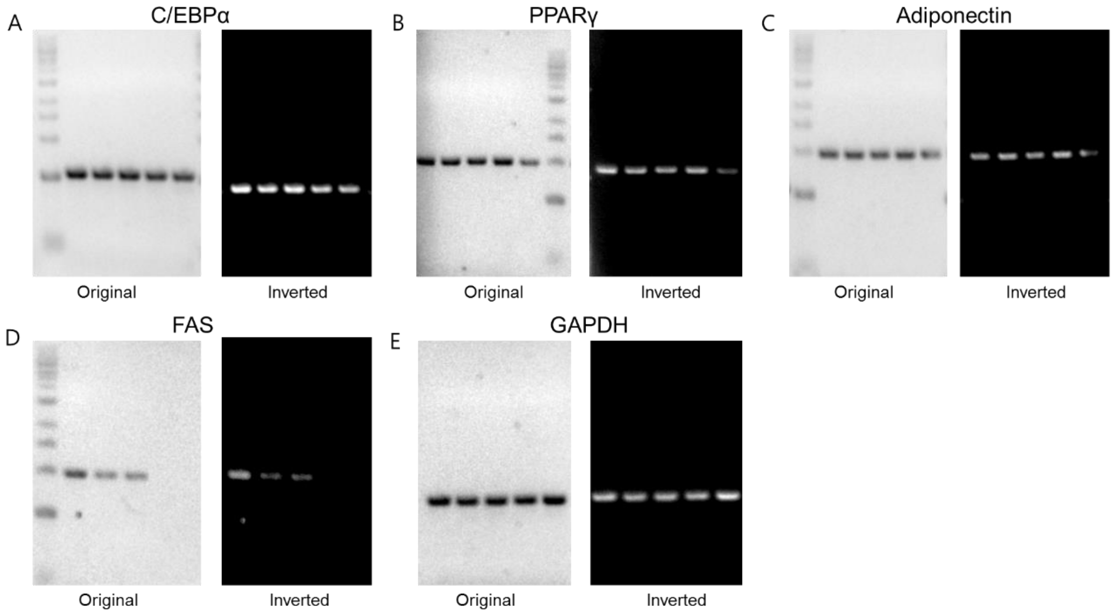

Figure S4. Full-length gel images corresponding to Fig 2E (C/EBPα, PPARγ, Adiponectin, FAS, and GAPDH).

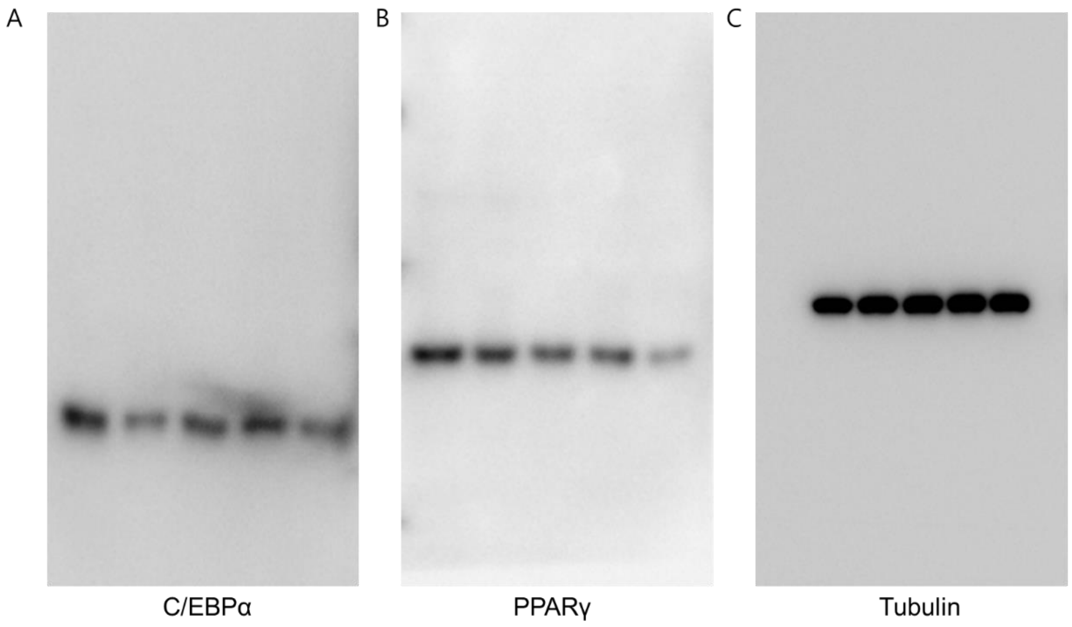

Figure S5. High resolution of full-length membrane blots corresponding to Fig 2D (C/EBPα, PPARγ, and Tubulin).

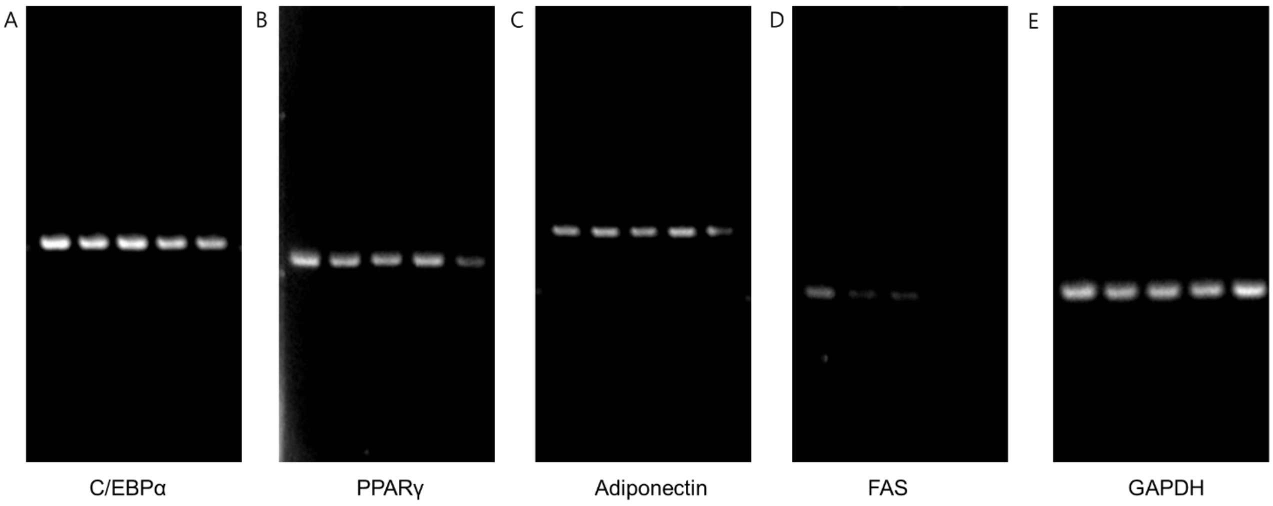

Figure S6. High resolution of full-length gel images corresponding to Fig 2E (C/EBPα, PPARγ, Adiponectin, FAS, and GAPDH).

Synthesis of isoquinolinoquinazolinones

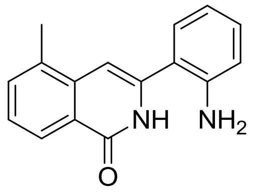

3-(2-aminophenyl)-5-methylisoquinolin-1(*2H*)-one (**7b**). The procedure used to prepare compound **7a** was carried out using compound *N,N*-diethyl-2,3-dimethyl-benzamide **5b** (2 g, 9.74 mmol), **6a** (1.76 g, 14.6 mmol), and *n*-BuLi (2.5 M solution in hexane; 12 mL) to afford compound **7b** as a yellow solid (515 mg, 21%). Mp: 216.9–218.1 °C. ^{1}H NMR (500 MHz, DMSO-d_6) δ: 11.21 (bs, 1H), 8.05 (d, J = 7.5 Hz, 1H), 7.52 (d, J = 6.0 Hz, 1H), 7.34 (t, J = 7.5 Hz, 1H), 7.14–7.09 (m, 2H), 6.76 (d, J = 7.5 Hz, 1H), 6.62 (t, J = 7.5 Hz, 1H), 6.59 (s, 1H), 5.20 (bs, 2H), 2.48 (s, 3H). MS (ESI) *m/z* = 251 $(M + H)^+$. Anal. Calcd. for $C_{16}H_{14}N_2O$•1.85 CH_2Cl_2•0.2 C_3H_7NO: C, 52.51; H, 4.56; N, 7.30. Found: C, 52.43; H, 4.45; N, 7.43.

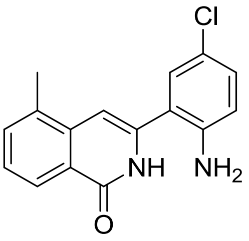

3-(2-amino-5-chlorophenyl)-5-methylisoquinolin-1(*2H*)-one (**7c**). The procedure used to prepare compound **7a** was used with compound **5b** (750 mg, 3.65 mmol), **6b** (557 mg, 3.65 mmol), and *n*-BuLi (2.5 M solution in hexane; 5 mL) to afford compound **7c** as a yellow solid (305 mg, 29%). Mp: 193.7–194.3 °C. ^{1}H NMR (300 MHz, DMSO-d_6) δ: 11.25 (bs, 1H), 8.05 (d, J = 8.1 Hz, 1H), 7.53 (d, J = 6.6 Hz, 1H), 7.55 (t, J = 7.8 Hz, 1H), 7.15–7.12 (m, 2H), 6.77–6.74 (m, 1H), 6.60 (s, 1H), 5.38 (bs, 2H), 2.48 (s, 3H). MS (ESI) *m/z* = 285 $(M + H)^+$. Anal. Calcd. for $C_{15}H_{12}N_2O$•0.15 $C_4H_8O_2$: C, 66.92; H, 4.80; N, 9.40. Found: C, 67.00; H, 4.92; N, 9.33.

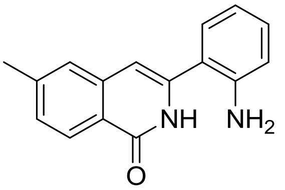

3-(2-aminophenyl)-6-methylisoquinolin-1(*2H*)-one (**7d**). The procedure used to prepare compound **7a** was carried out using compound *N,N*-diethyl-2,4-dimethyl-benzamide **5c** (1 g, 4.88 mmol), **6a** (472 mg, 4.0 mmol), and *n*-BuLi (2.5 M solution in hexane; 5 mL) to afford compound **7d** as a yellow solid (241 mg, 20%). Mp: 238.7–239.6 °C. ^{1}H NMR (300 MHz, DMSO-d$_6$) δ: 11.06 (bs, 1H), 8.06 (d, J = 8.1 Hz, 1H), 7.43 (s, 1H), 7.28 (dd, J = 8.1, 1.2 Hz, 1H), 7.12–7.07 (m, 2H), 6.77–6.74 (m, 1H), 6.61 (td, J = 7.8, 1.2 Hz, 1H), 6.49 (s, 1H), 5.13 (bs, 2H), 2.43 (s, 3H). MS (ESI) *m/z* = 251 (M + H)$^+$. Anal. Calcd. for $C_{16}H_{14}N_2O$•0.05 $C_4H_8O_2$: C, 75.37; H, 5.64; N, 10.82. Found: C, 75.49; H, 5.79; N, 10.62.

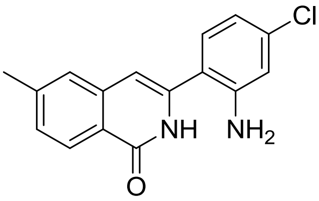

3-(2-amino-4-chlorophenyl)-6-methylisoquinolin-1(*2H*)-one (**7e**). The procedure used to prepare compound **7a** was carried out using compound **5c** (1 g, 4.88 mmol), **6c** (610 mg, 4.0 mmol), and *n*-BuLi (2.5 M solution in hexane; 5 mL) to afford compound **7e** as a yellow solid (237 mg, 17%). Mp: 236.5–237.8 °C. ^{1}H NMR (300 MHz, DMSO-d$_6$) δ: 11.11 (bs, 1H), 8.07 (d, J = 8.1 Hz, 1H), 7.43 (s, 1H), 7.29 (dd, J = 8.1, 1.2 Hz, 1H), 7.09 (d, J = 8.4 Hz, 1H), 6.79 (d, J = 2.4 Hz, 1H), 6.60 (dd, J = 8.4, 2.1 Hz, 1H), 6.47 (s, 1H), 5.47 (bs, 2H), 2.43 (s, 3H). MS (ESI) *m/z* = 285 (M + H)$^+$. Anal. Calcd. for $C_{16}H_{13}ClN_2O$•1.05 H_2O: C, 66.29; H, 5.30; N, 8.27. Found: C, 66.38; H, 5.44; N, 8.36.

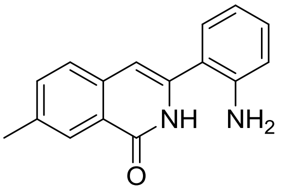

3-(2-aminophenyl)-7-methylisoquinolin-1(*2H*)-one (**7f**). The procedure used to prepare compound **7a** was carried out using compound *N,N*-diethyl-2,5-dimethyl-benzamide **5d** (1 g, 4.88 mmol), **6a** (472 mg, 4.0 mmol), and *n*-BuLi (2.5 M solution in hexane; 5 mL) to afford compound **7f** as an orange solid

(502 mg, 40%). Mp: 218.8–219.7 °C. ^{1}H NMR (300 MHz, DMSO-d$_6$) δ: 11.08 (bs, 1H), 7.99 (s, 1H), 7.56–7.49 (m, 2H), 7.12–7.07 (m, 2H), 6.77–6.74 (m, 1H), 6.61 (td, J = 7.5, 1.2 Hz, 1H), 6.53 (s, 1H), 5.12 (bs, 2H), 2.44 (s, 3H). Anal. MS (ESI) m/z = 251 (M + H)$^+$. Calcd. for $C_{16}H_{14}N_2O$•0.1 C_6H_{14}•0.05 CH_2Cl_2: C, 75.99; H, 5.94; N, 10.64. Found: C, 76.09; H, 5.76; N, 10.66.

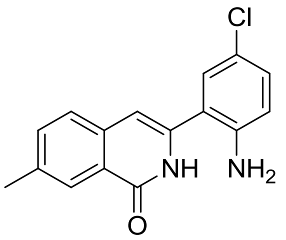

3-(2-amino-5-chlorophenyl)-7-methylisoquinolin-1(*2H*)-one (**7g**). The procedure used to prepare compound **7a** was carried out using compound **5d** (1 g, 4.88 mmol), **6b** (610 mg, 4.0 mmol), and *n*-BuLi (2.5 M solution in hexane; 5 mL) to afford compound **7g** as a yellow solid (550 mg, 25%). Mp: 232.1–233.0 °C. ^{1}H NMR (300 MHz, DMSO-d$_6$) δ: 11.15 (bs, 1H), 7.79 (s, 1H), 7.57–7.50 (m, 2H), 7.15–7.10 (m, 2H), 6.77–6.74 (m, 1H), 6.56 (s, 1H), 5.32 (bs, 2H), 2.45 (s, 3H). MS (ESI) m/z = 285 (M + H)$^+$.

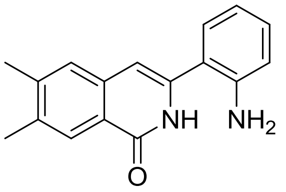

3-(2-aminophenyl)-6,7-dimethylisoquinolin-1(*2H*)-one (**7h**). The procedure used to prepare compound **7a** was carried out using compound *N,N*-diethyl-2,4,5-trimethyl-benzamide **5e** (1 g, 4.56 mmol), **6a** (539 mg, 4.56 mmol), and *n*-BuLi (2.5 M solution in hexane; 5 mL) to afford compound **7h** as a yellow solid (308 mg, 25%). Mp: 253.6–255.8 °C. ^{1}H NMR (300 MHz, DMSO-d$_6$) δ: 10.98 (bs, 1H), 7.94 (s, 1H), 7.41 (s, 1H), 7.12–7.07 (m, 2H), 6.77–6.74 (m, 1H), 6.61 (td, J = 7.5, 0.9 Hz, 1H), 6.46 (s, 1H), 5.10 (bs, 2H), 2.36 (s, 3H), 2.35 (s, 3H). MS (ESI) m/z = 265 (M + H)$^+$. Anal. Calcd. for $C_{17}H_{16}N_2O$•0.05 $C_4H_8O_2$•0.1 CH_2Cl_2: C, 76.95; H, 6.04; N, 10.10. Found: C, 76.95; H, 6.12; N, 10.19.

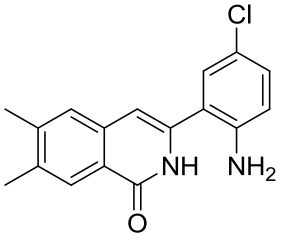

3-(2-amino-5-chlorophenyl)-6,7-dimethylisoquinolin-1(*2H*)-one (**7i**). The procedure used to prepare compound **7a** was carried out using compound **5e** (1 g, 4.56 mmol), **6b** (696 mg, 4.56 mmol), and *n*-BuLi (2.5 M solution in hexane; 5 mL) to afford compound **7i** as a yellow solid (660 mg, 48%). Mp: 272.8–273.7 °C. ^{1}H NMR (300 MHz, DMSO-d$_6$) δ: 11.04 (bs, 1H), 7.94 (s, 1H), 7.41 (s, 1H), 7.14–7.10 (m, 2H), 6.77–6.74 (m, 1H), 6.49 (s, 1H), 5.30 (bs, 2H), 2.36 (s, 3H), 2.35 (s, 3H). MS (ESI) *m/z* = 299 (M + H)$^+$. Anal. Calcd. for $C_{17}H_{15}ClN_2O$•0.6 H_2O: C, 65.96; H, 5.27; N, 9.05. Found: C, 65.90; H, 5.25; N, 9.00.

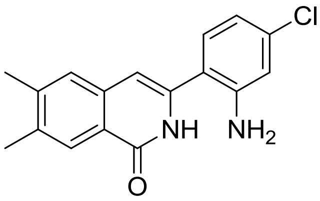

3-(2-amino-4-chlorophenyl)-6,7-dimethylisoquinolin-1(*2H*)-one (**7j**). The procedure used to prepare compound **7a** was carried out using compound **5e** (1 g, 4.56 mmol), **6c** (696 mg, 4.56 mmol), and *n*-BuLi (2.5 M solution in hexane; 5 mL) to afford compound **7j** as a yellow solid (320 mg, 23%). Mp: 273.6–274.7 °C. ^{1}H NMR (300 MHz, DMSO-d$_6$) δ: 11.03 (bs, 1H), 7.94 (s, 1H), 7.41 (s, 1H), 7.09 (d, J = 8.1 Hz, 1H), 6.79 (d, J = 2.1 Hz, 1H), 6.60 (dd, J = 8.1, 2.1 Hz, 1H), 6.45 (s, 1H), 5.44 (bs, 1H), 2.36 (s, 3H), 2.35 (s, 3H). MS (ESI) *m/z* = 299 (M + H)$^+$. Anal. Calcd. for $C_{17}H_{15}ClN_2O$•0.1 $C_4H_8O_2$•0.15 H2O: C, 67.35; H, 5.23; N, 9.03. Found: C, 67.35; H, 5.27; N, 8.99.

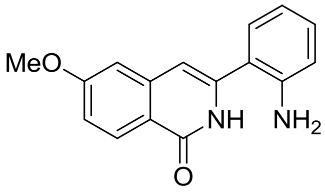

3-(2-aminophenyl)-6-methoxyisoquinolin-1(*2H*)-one (**7k**). The procedure used to prepare compound **7a** was carried out using compound *N,N*-diethyl-4-dimethoxy-2-methyl-benzamide **5f** (1 g, 4.52 mmol), **6a** (534 mg, 4.52 mmol), and *n*-BuLi (2.5 M solution in hexane; 5 mL) to afford compound **7k** as a yellow solid (296 mg, 24%). Mp: 209.1–209.9 °C. ^{1}H NMR (300 MHz, DMSO-d$_6$)

δ: 11.00 (bs, 1H), 8.09 (d, J = 9.0 Hz, 1H), 7.13–7.01 (m, 4H), 6.76 (dd, J = 9.0, 3.0 Hz, 1H), 6.64–6.39 (m, 1H), 6.50 (s, 1H), 5.13 (bs, 2H), 3.88 (s, 3H). MS (ESI) m/z = 267 (M + H)$^+$. Anal. Calcd. for $C_{16}H_{14}N_2O_2$•0.2 $C_4H_8O_2$•0.35 CH_2Cl_2: C, 67.29; H, 5.63; N, 8.94. Found: C, 67.31; H, 5.42; N, 8.96.

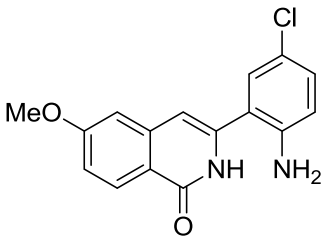

3-(2-amino-5-chlorophenyl)-6-methoxyisoquinolin-1(*2H*)-one (**7l**). The procedure used to prepare compound **7a** was carried out using compound **5f** (1 g, 4.52 mmol), **6b** (690 mg, 4.52 mmol), and *n*-BuLi (2.5 M solution in hexane; 5 mL) to afford compound **7l** as a yellow solid (275 mg, 20%). Mp: 238.4–239.9 °C. ^{1}H NMR (300 MHz, DMSO-d$_6$) δ: 8.09 (d, J = 9.0 Hz, 1H), 7.15–7.11 (m, 3H), 7.06–7.02 (m, 1H), 6.78–6.75 (m, 1H), 6.53 (s, 1H), 5.33 (bs, 2H), 3.86 (s, 3H). MS (ESI) m/z = 301 (M + H)$^+$.

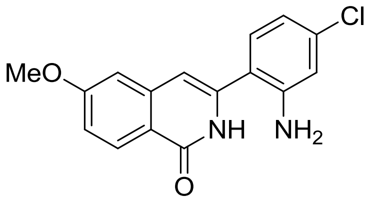

3-(2-amino-4-chlorophenyl)-6-methoxyisoquinolin-1(*2H*)-one (**7m**). The procedure used to prepare compound **7a** was carried out using compound **5f** (1 g, 4.52 mmol), **6c** (690 mg, 4.52 mmol), and *n*-BuLi (2.5 M solution in hexane; 5 mL) to afford compound **7m** as a yellow solid (380 mg, 28%). Mp: 252.2–253.0 °C. ^{1}H NMR (300 MHz, DMSO-d$_6$) δ: 11.02 (bs, 1H), 8.09 (d, J = 9.0 Hz, 1H), 7.13–7.02 (m, 3H), 6.79 (s, 1H), 6.61 (d, J = 6.0 Hz, 1H), 6.49 (s, 1H), 5.47 (bs, 2H), 3.86 (s, 3H). Anal. MS (ESI) m/z = 301 (M + H)$^+$. Calcd. for $C_{16}H_{13}ClN_2O_2$•0.35 $C_4H_8O_2$•0.15 CH_2Cl_2: C, 61.22; H, 4.71 N, 8.31. Found: C, 61.17; H, 4.94; N, 8.31.

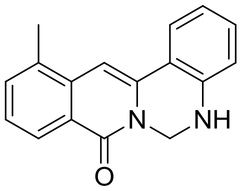

12-methyl-*5H*-isoquinolino[2,3-c]quinazolin-8(*6H*)-one (**8b**). The procedure used to prepare compound **8a** was carried out using compound **7b** (300 mg,

1.27 mmol), K_2CO_3 (438 mg, 3.17 mmol), and diiodomethane (1.36 g, 5.08 mmol) to afford compound **8b** as a yellow solid (185 mg, 55%). Mp: 226.5–228.1 °C. IR (cm^{-1}): 3296, 1588. ^{1}H NMR (500 MHz, DMSO-d$_6$) δ: 8.29 (d, J = 8.0 Hz, 1H), 7.82 (d, J = 8.0 Hz, 1H), 7.48 (d, J = 7.0 Hz, 1H), 7.34 (t, J = 8.0 Hz, 1H), 7.29–7.28 (m, 2H), 7.03–7.00 (m, 2H), 6.86 (d, J = 8.0 Hz, 1H), 5.36 (d, J = 3.0 Hz, 2H), 2.60 (s, 3H). ^{13}C NMR (125 MHz, DMSO-d$_6$) δ: 159.7, 144.5, 134.1, 134.1, 133.3, 130.1, 125.0, 123.3, 119.1, 117.5, 97.6, 52.2, 18.9. MS (ESI) m/z = 263 (M + H)$^+$. Anal. Calcd. for $C_{17}H_{14}N_2O$•0.05 $C_4H_8O_2$: C, 77.46; H, 5.44; N, 10.50. Found: C, 77.43; H, 5.41; N, 10.32. HPLC: t_r 2.28 min, purity 98.9%.

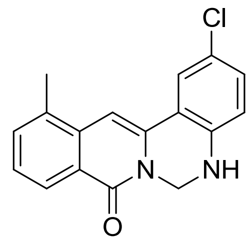

2-chloro-12-methyl-5*H*-isoquinolino[2,3-c]quinazolin-8(6*H*)-one (**8c**). The procedure used to prepare compound **8a** was carried out using compound **7c** (280 mg, 0.98 mmol), K_2CO_3 (387 mg, 2.80 mmol), and diiodomethane (1.20 g, 4.47 mmol) to afford compound **8c** as a yellow solid (60 mg, 21%). Mp: 240.5–242.1 °C. IR (cm^{-1}): 3263, 1592. ^{1}H NMR (300 MHz, DMSO-d$_6$) δ: 8.12 (d, J = 2.4 Hz, 1H), 8.08 (d, J = 8.1 Hz, 1H), 7.56–7.54 (m, 1H), 7.36 (t, J = 7.8 Hz, 1H), 7.27 (dd, J = 8.4, 2.4 Hz, 1H), 7.18 (s, 1H), 6.99 (bs, 1H), 6.92 (d, J = 8.7 Hz, 1H), 5.14 (d, J = 2.1 Hz, 2H), 2.60 (s, 3H). ^{13}C NMR (125 MHz, DMSO-d$_6$) δ: 160.2, 144.9, 134.6, 134.5, 133.7, 130.5, 125.4, 123.7, 119.5, 117.9, 98.0, 52.6, 19.3. MS (ESI) m/z = 297 (M + H)$^+$. Anal. Calcd. for $C_{17}H_{13}ClN_2O$•0.1 H_2O•0.1 $C_4H_8O_2$: C, 67.99; H, 4.59; N, 9.11. Found: C, 68.13; H, 4.41; N, 8.95. HPLC: t_r 2.45 min, purity 95.4%.

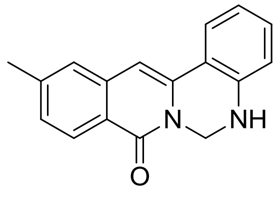

11-methyl-5*H*-isoquinolino[2,3-c]quinazolin-8(6*H*)-one (**8d**). The procedure used to prepare compound **8a** was carried out using compound **7d** (218 mg, 0.87 mmol), K_2CO_3 (301 mg, 2.18 mmol), and diiodomethane

(933 mg, 3.48 mmol) to afford compound **8d** as a yellow solid (97 mg, 42%). Mp: 119.1–120.4 °C. IR (cm^{-1}): 3300, 1598. ^1H NMR (300 MHz, DMSO-d_6) δ: 8.09 (d, J = 8.4 Hz, 1H), 7.84 (d, J = 7.5 Hz, 1H), 7.47 (s, 1H), 7.29–7.22 (m, 2H), 7.08 (s, 1H), 6.92–6.86 (m, 2H), 6.82 (bs, 1H), 5.12 (d, J = 2.1 Hz, 2H), 2.44 (s, 3H). ^{13}C NMR (125 MHz, CDCl3) δ: 160.8, 144.1, 142.9, 137.0, 135.4, 130.3, 127.8, 124.6, 121.0, 119.3, 116.7, 100.9, 52.9, 21.8. MS (ESI) m/z = 263 $(M + H)^+$. Anal. Calcd. for $C_{17}H_{14}N_2O$•0.1 C_6H_{14}•0.1 CH_2Cl_2: C, 76.08; H, 5.63; N, 10.03. Found: C, 76.33; H, 5.34; N, 10.09. HPLC: t_r 2.19 min, purity 98.5%.

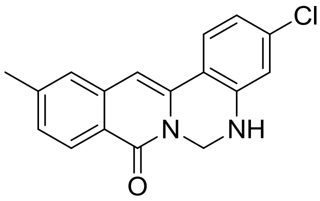

3-chloro-11-methyl-*5H*-isoquinolino[2,3-c]quinazolin-8(*6H*)-one (**8e**). The procedure used to prepare compound **8a** was carried out using compound **7e** (207 mg, 0.73 mmol), K_2CO_3 (251 mg, 1.82 mmol), and diiodomethane (779 mg, 2.91 mmol) to afford compound **8e** as a yellow solid (96 mg, 44%). Mp: 210.0–211.5 °C. IR (cm^{-1}): 3329, 1594. ^1H NMR (300 MHz, DMSO-d_6) δ: 8.09 (d, J = 8.1 Hz, 1H), 7.86 (d, J = 8.4 Hz, 1H), 7.47 (s, 1H), 7.30 (d, J = 8.4 Hz, 1H), 7.12 (s, 1H), 7.05 (s, 1H), 6.95–6.89 (m, 2H), 5.14 (d, J = 2.1 Hz, 2H), 2.44 (s, 3H). ^{13}C NMR (125 MHz, DMSO-d_6) δ: 159.5, 146.7, 142.8, 136.8, 134.8, 134.7, 127.9, 127.1, 126.4, 126.0, 121.9, 119.2, 116.2, 115.0, 99.8, 52.0, 21.3. MS (ESI) m/z = 297 $(M + H)^+$. Anal. Calcd. for $C_{17}H_{13}ClN_2O$•0.05 C_6H_{14}•0.1 CH_2Cl_2: C, 67.51; H, 4.53; N, 9.05. Found: C, 67.77; H, 4.40; N, 9.02. HPLC: t_r 2.47 min, purity 95.6%.

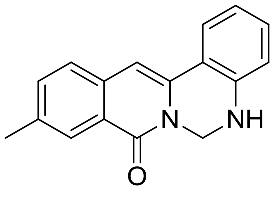

10-methyl-5H-isoquinolino[2,3-c]quinazolin-8(6H)-one (**8f**). The procedure used to prepare compound **8a** was carried out using compound **7f** (80 mg, 0.32 mmol), K_2CO_3 (110 mg, 0.80 mmol), and diiodomethane

(342 mg, 1.28 mmol) to afford compound **8f** as a yellow solid (56 mg, 67%). Mp: 213.2–214.5 °C. IR (cm^{-1}): 3290, 1593. ^1H NMR (300 MHz, DMSO-d_6) δ: 8.01 (s, 1H), 7.83 (d, J = 7.2 Hz, 1H), 7.60 (d, J = 8.1 Hz, 1H), 7.53 (dd, J = 8.1, 1.8 Hz, 1H), 7.23 (td, J = 8.1, 1.2 Hz, 1H), 7.13 (s, 1H), 6.91–6.86 (m, 2H), 6.80 (bs, 1H), 5.13 (d, J = 2.1 Hz, 2H), 2.44 (s, 3H).). ^{13}C NMR (125 MHz, DMSO-d_6) δ: 159.6, 145.5, 135.7, 134.8, 134.6, 134.0, 130.3, 126.6, 126.5, 124.6, 123.9, 119.5, 117.6, 115.9, 99.7, 52.3, 21.1. MS (ESI) *m/z* = 263 $(M + H)^+$. Anal. Calcd. for $C_{17}H_{14}N_2O$•0.05 H_2O•0.05 $C_4H_8O_2$: C, 77.20; H, 5.46; N, 10.47. Found: C, 77.29; H, 5.38; N, 10.28. HPLC: t_r 2.21 min, purity 99.0%.

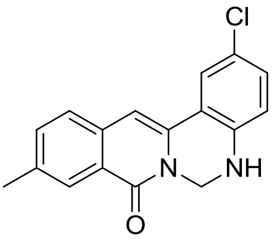

2-chloro-10-methyl-*5H*-isoquinolino[2,3-c]quinazolin-8(*6H*)-one (**8g**). The procedure used to prepare compound **8a** was carried out using compound **7g** (200 mg, 0.70 mmol), K_2CO_3 (242 mg, 1.75 mmol), and diiodomethane (752 mg, 2.81 mmol) to afford compound **8g** as a yellow solid (80 mg, 38%). Mp: 202.6–205.1 °C. IR (cm^{-1}): 3299, 1598. ^1H NMR (300 MHz, DMSO-d_6) δ: 8.01 (d, J = 0.9 Hz, 1H), 7.90 (d, J = 2.4 Hz, 1H), 7.60 (d, J = 8.1 Hz, 1H), 7.54 (dd, J = 8.1, 1.8 Hz, 1H), 7.27–7.24 (m, 2H), 6.97 (bs, 1H), 6.90 (d, J = 8.4 Hz, 1H), 5.13 (d, J = 2.4 Hz, 2H), 2.44 (s, 3H).). ^{13}C NMR (125 MHz, DMSO-d_6) δ: 159.4, 144.2, 136.1, 134.1, 133.5, 129.8, 126.6, 124.2, 123.8, 123.2, 119.0, 117.5, 100.6, 52.2, 21.1. MS (ESI) *m/z* = 297 $(M + H)^+$. Anal. Calcd. for $C_{17}H_{13}ClN_2O$•0.05 $C_4H_8O_2$•0.3 CH_2Cl_2: C, 64.35; H, 4.32; N, 8.58. Found: C, 64.22; H, 4.21; N, 8.47. HPLC: t_r 2.52 min, purity 96.6%.

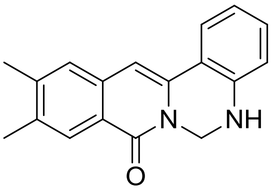

10,11-dimethyl-*5H*-isoquinolino[2,3-c]quinazolin-8(*6H*)-one (**8h**). The procedure used to prepare compound **8a** was carried out using compound **12h** (248 mg, 0.94 mmol), K_2CO_3 (324 mg, 2.34 mmol), and

diiodomethane (1 g, 3.76 mmol) to afford compound **8h** as a yellow solid (120 mg, 46%). Mp: 212.7–214.2 °C. IR (cm^{-1}): 3302, 1593. ^{1}H NMR (300 MHz, DMSO-d$_6$) δ: 7.97 (s, 1H), 7.84–7.81 (m, 1H), 7.46 (s, 1H), 7.26–7.23 (m, 1H), 7.05 (s, 1H), 6.91–6.84 (m, 2H), 6.78 (bs, 1H), 5.12 (d, J = 3.0 Hz, 2H), 2.36 (s, 3H), 2.34 (s, 3H).). ^{13}C NMR (125 MHz, DMSO-d$_6$) δ: 159.4, 145.4, 142.2, 135.3, 135.0, 134.9, 130.2, 127.0, 126.5, 124.5, 122.2, 119.5, 117.6, 115.9, 99.4, 52.2, 19.8, 19.5. MS (ESI) *m/z* = 277 (M + H)$^+$. Anal. Calcd. for $C_{18}H_{16}N_2O{\bullet}0.1\ C_4H_8O_2$: C, 77.50; H, 5.94; N, 9.82. Found: C, 77.60; H, 5.82; N, 9.68. HPLC: t_r 2.37 min, purity 97.0%.

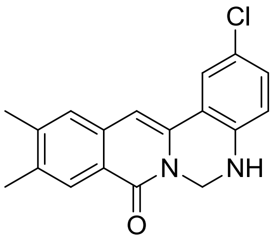

2-chloro-10,11-dimethyl-5*H*-isoquinolino[2,3-c]quinazolin-8(*6H*)-one (**8i**). The procedure used to prepare compound **8a** was carried out using compound **7i** (496 mg, 1.66 mmol), K_2CO_3 (573 mg, 4.15 mmol), and diiodomethane (1.78 g, 6.64 mmol) to afford compound **8i** as a yellow solid (94 mg, 18%). Mp: 268.7–270.6 °C. IR (cm^{-1}): 3259, 1592. ^{1}H NMR (300 MHz, DMSO-d$_6$) δ: 7.97 (s, 1H), 7.90 (d, J = 3.0 Hz, 1H), 7.45 (s, 1H), 7.25 (dd, J = 9.0, 3.0 Hz, 1H), 7.18 (s, 1H), 6.95 (bs, 1H), 6.90 (d, J = 9.0 Hz, 1H), 5.14 (d, J = 3.0 Hz, 2H), 2.36 (s, 3H), 2.35 (s, 3H). ^{13}C NMR (125 MHz, DMSO-d$_6$) δ: 159.4, 144.3, 142.4, 135.9, 134.9, 133.6, 129.8, 126.8, 123.9, 122.5, 119.2, 117.6, 100.5, 52.2, 19.9, 19.6. MS (ESI) *m/z* = 311 (M+H)$^+$. Anal. Calcd. for $C_{18}H_{15}N_2O{\bullet}0.1\ H_2O{\bullet}0.15\ C_4H_8O_2$: C, 68.57; H, 5.07; N, 8.60. Found: C, 68.66; H, 4.92; N, 8.46. HPLC: t_r 2.72 min, purity 98.1%.

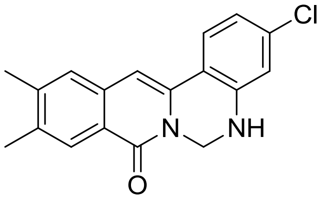

3-chloro-10,11-dimethyl-5*H*-isoquinolino[2,3-c]quinazolin-8(*6H*)-one (**8j**). The procedure used to prepare compound **8a** was carried out using compound **7j** (260 mg, 0.87 mmol), K_2CO_3 (300 mg, 2.17

mmol), and diiodomethane (932 mg, 3.48 mmol) to afford compound **8j** as a yellow solid (149 mg, 55%). Mp: 235.1–236.4 °C. IR (cm^{-1}): 3326, 1596. ^{1}H NMR (300 MHz, DMSO-d$_6$) δ: 7.96 (s, 1H), 7.85 (d, J = 9.0 Hz, 1H), 7.45 (s, 1H), 7.08 (s, 1H), 7.02 (bs, 1H), 6.94–6.88 (m, 2H), 5.14 (d, J = 3.0 Hz, 2H), 2.36 (s, 3H), 2.35 (s, 3H). ^{13}C NMR (125 MHz, DMSO-d$_6$) δ: 159.4, 146.6, 142.3, 135.6, 134.9, 134.4, 134.0, 127.0, 126.6, 126.2, 122.2, 119.2, 116.6, 114.9, 99.7, 52.0, 19.8, 19.5. MS (ESI) m/z = 311 (M + H)$^+$. Anal. Calcd. for $C_{18}H_{15}ClN_2O$•0.15 $C_4H_8O_2$: C, 68.95; H, 5.04; N, 8.65. Found: C, 69.10; H, 4.89; N, 8.58. HPLC: t$_r$ 2.75 min, purity 95.0%.

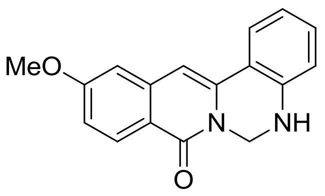

11-methoxy-*5H*-isoquinolino[2,3-c]quinazolin-8(*6H*)-one (**8k**). The procedure used to prepare compound **8a** was carried out using compound **7k** (220 mg, 0.83 mmol), K_2CO_3 (285 mg, 2.06 mmol), and diiodomethane (889 mg, 3.32 mmol) to afford compound **8k** as a yellow solid (120 mg, 52%). Mp: 145.4–148.0 °C. IR (cm^{-1}): 3265, 1577. ^{1}H NMR (300 MHz, DMSO-d$_6$) δ: 8.10 (d, J = 9.0 Hz, 1H), 7.82 (dd, J = 9.0, 3.0 Hz, 1H), 7.28–7.22 (m, 1H), 7.13 (d, J = 6.0 Hz, 1H), 7.09 (d, J = 3.0 Hz, 1H), 6.92–6.87 (m, 2H), 6.82 (bs, 1H), 5.11 (d, J = 3.0 Hz, 2H), 3.88 (s, 3H). ^{13}C NMR (125 MHz, DMSO-d$_6$) δ: 162.8, 159.7, 146.1, 139.4, 136.7, 130.9, 129.6, 125.0, 119.9, 117.8, 116.4, 116.1, 107.5, 99.9, 55.9, 55.9, 52.5. MS (ESI) m/z = 279 (M + H)$^+$. Anal. Calcd. for $C_{17}H_{14}N_2O_2$•0.05 $C_4H_8O_2$•CH_2Cl_2: C, 72.20; H, 5.09; N, 9.76. Found: C, 72.19; H, 5.00; N, 9.67. HPLC: t$_r$ 2.02 min, purity 97.0%.

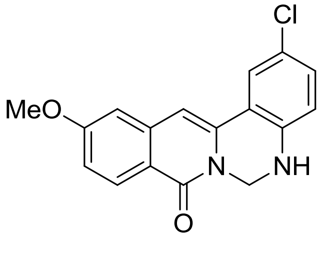

2-chloro-11-methoxy-*5H*-isoquinolino[2,3-c]quinazolin-8(*6H*)-one (**8l**). The procedure used to prepare compound **8a** was carried out

using compound **7l** (203 mg, 0.67 mmol), K_2CO_3 (233 mg, 1.69 mmol), and diiodomethane (718 mg, 2.68 mmol) to afford compound **8l** as a yellow solid (50 mg, 24%). Mp: 213.6–215.1 °C. IR (cm^{-1}): 3275, 1561. ^1H NMR (300 MHz, DMSO-d_6) δ: 8.11 (d, J = 6.0 Hz, 1H), 7.88 (s, 1H), 7.27 (dd, J = 9.0, 3.0 Hz, 1H), 7.23 (s, 1H), 7.14 (d, J = 3.0 Hz, 1H), 7.08–6.90 (m, 3H), 5.11 (d, J = 3.0 Hz, 2H), 3.88 (s, 3H). ^{13}C NMR (125 MHz, DMSO-d_6) δ: 162.4, 159.2, 144.4, 138.7, 134.8, 130.0, 129.1, 123.9, 123.1, 118.7, 118.0, 117.6, 116.0, 107.3, 100.4, 55.4, 51.9. MS (ESI) m/z = 313 (M + H)$^+$. Anal. Calcd. for $C_{17}H_{13}ClN_2O_2$•0.1 $C_4H_8O_2$•0.15 CH_2Cl_2: C, 63.05; H, 4.25; N, 8.38. Found: C, 63.14; H, 4.15; N, 8.21. HPLC: t_r 2.15 min, purity 97.5%.

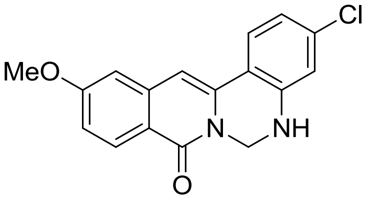

3-chloro-11-methoxy-*5H*-isoquinolino[2,3-c]quinazolin-8(*6H*)-one (**8m**). The procedure used to prepare compound **8a** was carried out using compound **7m** (215 mg, 0.71 mmol), K_2CO_3 (247 mg, 1.79 mmol), and diiodomethane (761 mg, 2.84 mmol) to afford compound **8m** as a yellow solid (97 mg, 44%). Mp: 203.9–205.2 °C. IR (cm^{-1}): 3321, 1591. ^1H NMR (300 MHz, DMSO-d_6) δ: 8.11 (d, J = 9.0 Hz, 1H), 7.85–7.82 (m, 1H), 7.14–7.13 (m, 2H), 7.06–7.03 (m, 2H), 6.95–6.89 (m, 2H), 5.12 (d, J = 3.0 Hz, 2H), 3.88 (s, 3H). ^{13}C NMR (125 MHz, DMSO-d_6) δ: 162.4, 159.2, 146.8, 138.7, 135.3, 134.8, 129.2, 126.3, 119.2, 117.9, 116.1, 115.9, 115.0, 107.2, 99.8, 55.4, 51.8. MS (ESI) m/z = 313 (M + H)$^+$. Anal. Calcd. for $C_{17}H_{13}ClN_2O_2$•0.1 $C_4H_8O_2$•0.1 CH_2Cl_2: C, 63.68; H, 4.28; N, 8.49. Found: C, 63.82; H, 4.11; N, 8.43. HPLC: t_r 2.17 min, purity 98.4%.

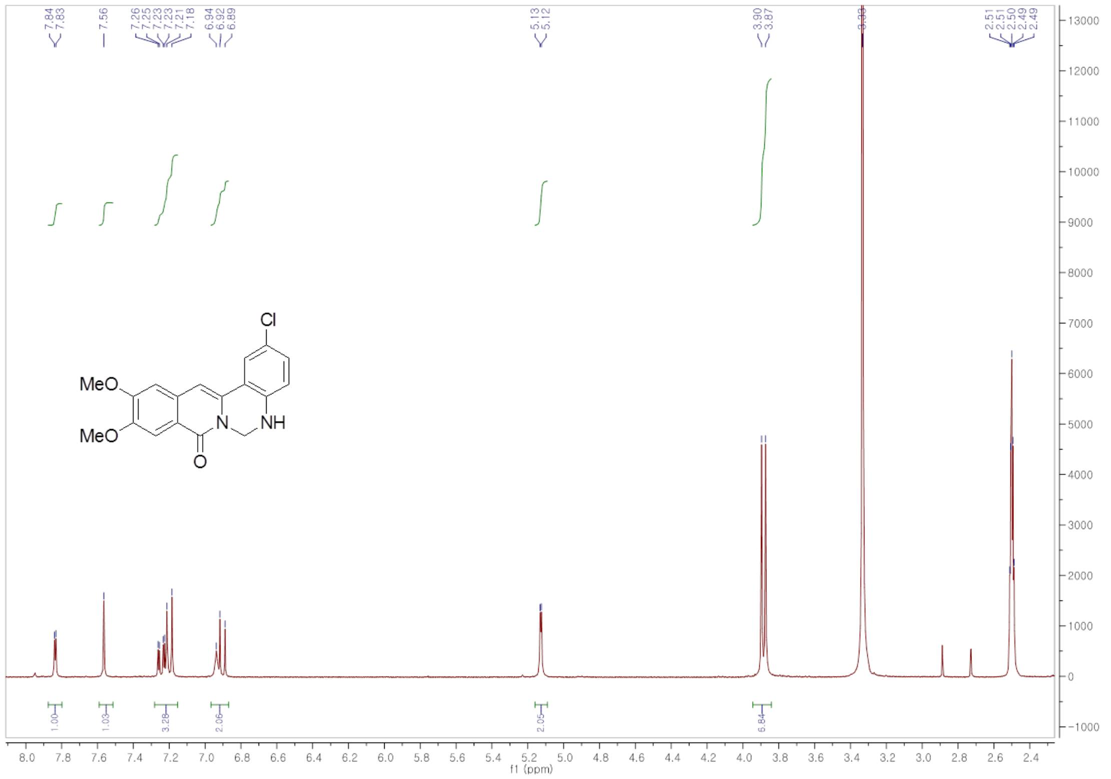

Figure S3. ^{1}**H NMR (300 MHz, DMSO-d_6) spectrum of 8n.**

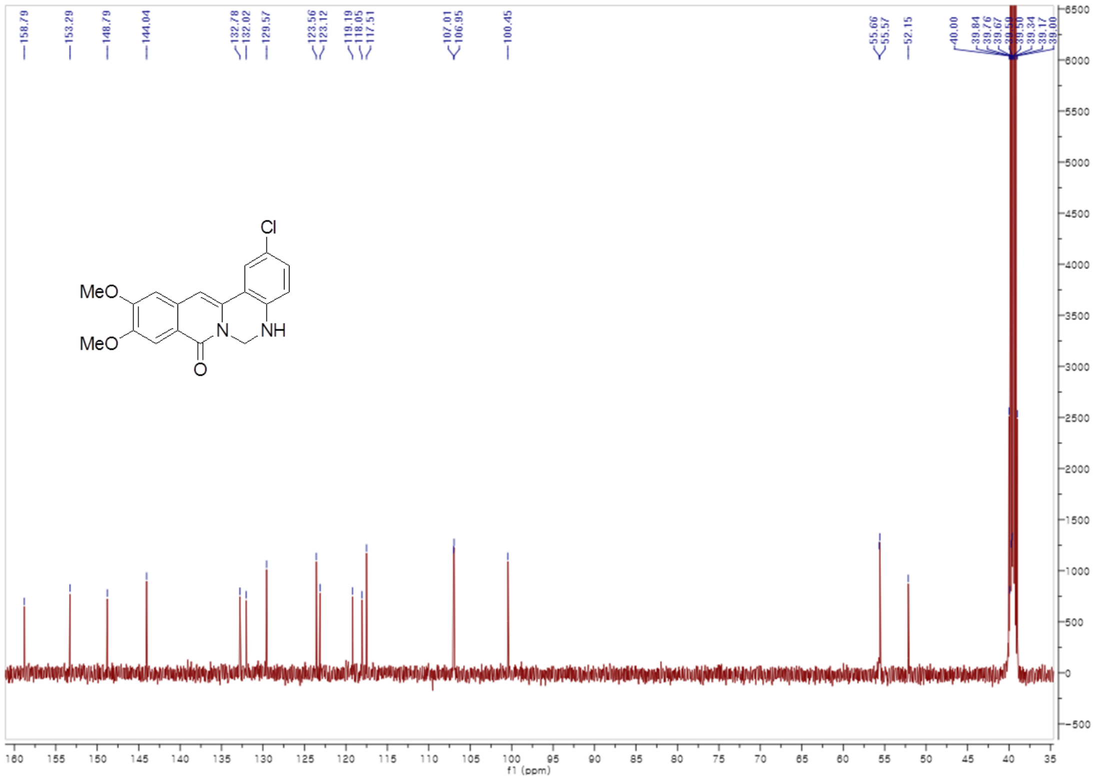

Figure S4. ^{13}**C NMR (125 MHz, DMSO-d_6) spectrum of 8n.**

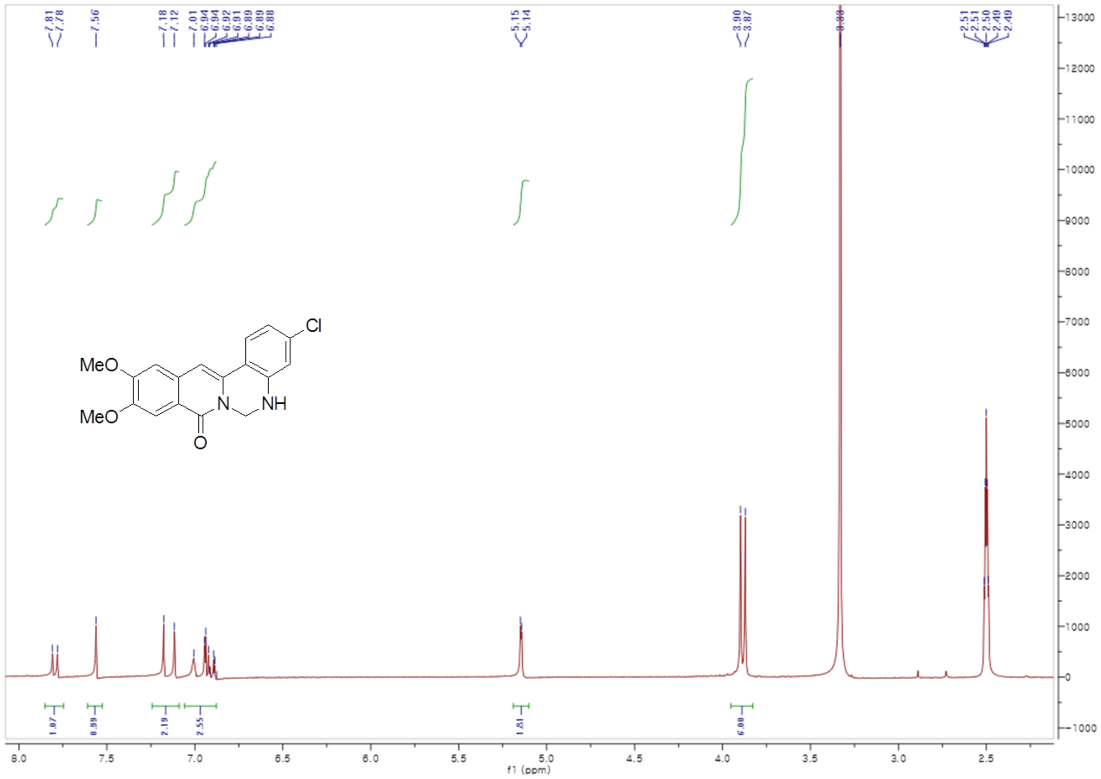

Figure S5. ^{1}H NMR (300 MHz, DMSO-d_6) spectrum of **8o.**

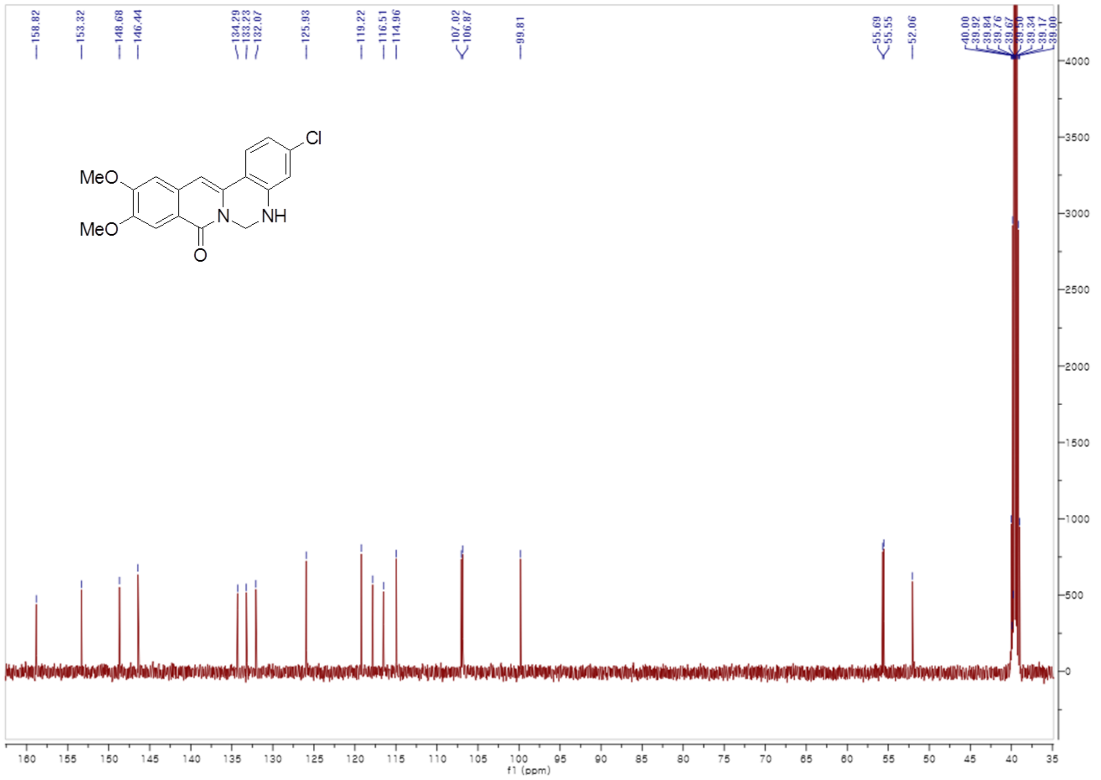

Figure S6. ^{13}C NMR (125 MHz, DMSO-d_6) spectrum of **8o.**
